# Generating normative data from web-based administration of the Cambridge Neuropsychological Test Automated Battery using a Bayesian framework

**DOI:** 10.3389/fdgth.2024.1294222

**Published:** 2024-09-20

**Authors:** Elizabeth Wragg, Caroline Skirrow, Pasquale Dente, Jack Cotter, Peter Annas, Milly Lowther, Rosa Backx, Jenny Barnett, Fiona Cree, Jasmin Kroll, Francesca Cormack

**Affiliations:** ^1^ Clinical Science, Cambridge Cognition, Cambridge, United Kingdom; ^2^ School of Psychological Science, University of Bristol, Bristol, United Kingdom; ^3^ Research & Development, Lundbaek, Copenhagen, Denmark; ^4^ Institute of Cognitive Neuroscience, University College London, London, United Kingdom; ^5^ Department of Psychiatry, University of Cambridge, Cambridge, United Kingdom

**Keywords:** normative data, cognition, neuropsychology, ageing, Bayesian statistics

## Abstract

**Introduction:**

Normative cognitive data can distinguish impairment from healthy cognitive function and pathological decline from normal ageing. Traditional methods for deriving normative data typically require extremely large samples of healthy participants, stratifying test variation by pre-specified age groups and key demographic features (age, sex, education). Linear regression approaches can provide normative data from more sparsely sampled datasets, but non-normal distributions of many cognitive test results may lead to violation of model assumptions, limiting generalisability.

**Method:**

The current study proposes a novel Bayesian framework for normative data generation. Participants (*n* = 728; 368 male and 360 female, age 18–75 years), completed the Cambridge Neuropsychological Test Automated Battery via the research crowdsourcing website Prolific.ac. Participants completed tests of visuospatial recognition memory (Spatial Working Memory test), visual episodic memory (Paired Associate Learning test) and sustained attention (Rapid Visual Information Processing test). Test outcomes were modelled as a function of age using Bayesian Generalised Linear Models, which were able to derive posterior distributions of the authentic data, drawing from a wide family of distributions. Markov Chain Monte Carlo algorithms generated a large synthetic dataset from posterior distributions for each outcome measure, capturing normative distributions of cognition as a function of age, sex and education.

**Results:**

Comparison with stratified and linear regression methods showed converging results, with the Bayesian approach producing similar age, sex and education trends in the data, and similar categorisation of individual performance levels.

**Conclusion:**

This study documents a novel, reproducible and robust method for describing normative cognitive performance with ageing using a large dataset.

## Introduction

Well-validated computerised neuropsychological tests such as the Cambridge Neuropsychological Test Automated Battery (CANTAB) are widely used but require in-person assessments entailing significant costs and time. Granular data from the web-based version of the CANTAB may pioneer the way neuropsychological tests are conducted, becoming an integral part of clinical care and large-scale research trials. Advantages of web-based assessments include test standardization, precise response measurements and have shown higher response rates compared to supervised administration (1, 2). Additionally, online assessments improve reach to specialised and typically underrepresented populations, are cost and time-effective and permit flexibility in timing and location (3, 4).

CANTAB performance indices are satisfactorily comparability between web-based and in-person assessments (5). However, the integration of remote, web-based adaptations of existing tests require new statistical norms (6) which are valuable as reference data for identifying impairments and age-related declines (7). One approach to deriving normative data is through grouping test performance for specific age-ranges often spanning multiple years (8). However, this approach may not provide the required detail to observe year-on-year changes, such as more rapid declines in cognitive function seen in older age (9). Norms derived through linear regression can generate a year-by-year view of age-related change. However, this approach may prove less sensitive to identifying higher levels of impairment, particularly at age extremes in the population where data is often sparser (10).

Bayesian approaches for establishing performance relative to normative data are more accommodating to non-normal distributions and can incorporate uncertainty introduced by ties within the data, where the same test score is obtained by more than one person (11). Non-normal test distributions are common in cognitive assessments (12), where for error-count response variables frequently include an excess of zeroes, and ties are inevitable for tasks with a limited number of responses choices. In case studies where individual performance is compared to a normative group, point and interval estimates of percentile norms typically show a good degree of convergence with classical frequentist methods (11, 13).

The current study describes a novel methodological approach for generating normative cognitive data from the CANTAB administered via the internet. A large cognitive dataset is analysed using Bayesian statistical methods to generate a large synthetic normative dataset capturing the normative processes of cognition as a function of age, sex and education. As such the aims of the current study are to (1) describe this approach and methodology for providing robust estimations of performance percentiles taking into consideration age, sex and education; (2) describe cognitive performance across age, sex and education using these methods and (3) examine sensitivity of this novel approach in comparison to other methods for deriving normative data.

## Methods

### Participants

Data was collected using a web-based cognitive assessment application between September 2017 and April 2018. Participants were recruited using Prolific, an online crowdsourcing platform for advertising web-based studies (14). Previous research has shown adequate data quality on this platform, and better than other available platforms (15). To be included in the study, participants had to meet the following eligibility criteria: aged ≥18 years, fluent English speaker, no history of head injury resulting in a loss of consciousness, not diagnosed with a mental health condition that is uncontrolled (by medication or intervention) and which has a significant impact on daily life, never diagnosed with mild cognitive impairment or dementia.

### Procedure

After logging into Prolific, participants clicked on a link to the study homepage, which provided a detailed explanation of the study. Participants were asked to provide basic demographic information including their age, sex and highest level of education. Level of education was entered as follows: (1) left formal education before age 16, (2) left formal education at age 16, (3) left formal education at age 18, (4) undergraduate degree/higher national diploma, (5) Master's degree/postgraduate diploma, (6) PhD. Information on country of origin and country of residence was obtained from Prolific Academic participant databases.

**Figure 1:**
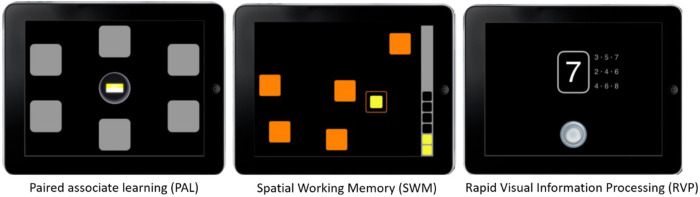
Stills of tests administered as would be displayed on an iPad.

Participants were instructed to turn on the sound on their device and to complete the study on their own, in a quiet room and to the best of their ability. They were instructed not to participate under the influence of alcohol or other substances, or if they were feeling unusually stressed, tired, or unwell. They were then asked to complete three non-verbal cognitive tests, taking approximately 30 min. Assessments were delivered via the CANTAB web-based testing application, which displayed tests visually on participants’ devices, and provided instructions via voiceover (Figure 1).

Participants responded via touch screen or mouse/trackpad click depending on the response modality of their own devices. The CANTAB battery consists of a suite of nineteen language-independent cognitive tests. Three tests were initially selected for validating the Bayesian methodology for deriving normative data. Cognitive tests and outcome measures are described below. Further information on each test can be found on the Cambridge Cognition website (http://www.cambridgecognition.com/cantab/cognitive-tests/). All subjects provided informed consent prior to their participation and were reimbursed £2.50 for their time on completion.

### Measures

• Paired Associate Learning (16) (PAL) is a test of visual episodic memory lasting eight minutes (Figure 1). During this task, a number of boxes are displayed around the device screen. The interior of each box is revealed in a randomised order and some boxes contain a pattern. After the interior of each box is shown, the same patterns are displayed in the middle of the screen one at a time and the participant must select the box in which the pattern was originally located. If the participant makes an error, the boxes are opened in sequence again to remind them of the pattern locations. Initially the test includes six boxes, in which there are two patterns. The task increases in difficulty after each stage is completed successfully, with trials including two, four, and six different patterns in six boxes, and finally eight different patterns in eight boxes, which the participant is then required to locate. Where a participant fails to identify the location of patterns successfully after four attempts the task terminates. Key outcome measures include PAL Total Errors Adjusted (PALTEA), the total number of errors adjusted for the stages not completed due to early discontinuation (range = 0–70), and PAL First Attempt Memory Score (PALFAMS), the number of times a participant chooses the correct box on their first attempt across each stage (score range 0–20).
• Spatial Working Memory (17) (SWM) is a four minute test of retention and manipulation of visuospatial information. Participants find tokens in coloured boxes presented on the screen and move them to a collection area. The key task instruction is that tokens will not be in the same box twice in each trial. Outcome measures include SWM Between Errors (SWMBE): the number of times the participant incorrectly revisits a box, calculated across all assessed 4, 6 and 8 token trials (range of possible scores 0–153); and SWM Strategy (SWMS): the number of unique boxes from which a participant starts a new search in the 6 and 8 box trials (range of possible scores 2–14). More efficient searches are carried out by searching boxes in a fixed order.
• Rapid Visual Information Processing (18) (RVP) is a test of sustained attention lasting 7 min. Digits from 2 to 9 were presented successively at the rate of 100 digits per minute in pseudo-random order. Participants are asked to respond to target sequences of digits (for example, 2–4–6, 3–5–7, 4–6–8) as quickly as possible by clicking or pressing a button at the bottom centre of the device screen. Level of difficulty varies with either one- or three-target sequences that the participant must watch for at the same time. Outcome measures included a signal detection measure of response sensitivity to the target regardless of response tendency (RVP A’: expected range is 0 to 1), and probability of false alarm (RVPPFA: expected range 0 to 1)

## Statistical analysis

### Data preparation and cleaning

The six levels of education were collapsed into two categories, “high” - leaving school after age ≥ 16, and “low” - leaving school age < 16. Potential influence of distraction during task performance was examined by comparing individuals who completed CANTAB tasks on full-screen mode and those who did not. This was tested through graphical and distribution comparisons separated by age bands, sex and educational level, which showed no difference in test performance. Similarly, no differences were found in the graphical and distribution comparison of the Bayesian generalised linear models (GLM) in those using full-screen mode and the entire sample. All individuals were therefore included in the downstream analysis.

### Bayesian normative data generation

Using principles described previously (19, 20), a method for generating synthetic data was developed to preserve the statistical properties of the dataset. Bayesian methods allow prior knowledge about model parameters (e.g., sparsity, non-negativity) to be explicitly incorporated into statistical models (21). The models combine these priors with authentic data to create posterior distributions of the data under investigation. Using Markov Chain Monte Carlo (MCMC) algorithms it is possible to draw random samples of the posterior distribution, providing a synthetic dataset from which normative data can be derived. With this approach, we use our authentic data to inform generation of a synthetic dataset capturing normative process of cognition across age, sex and education, incorporating variability but not including extreme outliers.

Outcome measures from CANTAB tasks capture processes that do not follow a normal distribution. As a result, Bayesian GLMs were used to model CANTAB test performance as a function of age, as they incorporate a wide family of distributions representing these measures. The parameterisation of the response distribution allows the appropriate test structure to considered. For example, when using a test with error-count type responses, which often includes an excess of zeroes or ones in outcome data, it is important to fit mixed continuous-discrete distributions, such as zero- and one-inflated or hurdle models (22, 23). All Bayesian GLMs were developed in Stan code using the brms package (24) in a reproducible R environment (version 3.4.4) with version control using git (version 2.15) in a standalone Docker container (version 18.03).

Keeping the default brms prior, a half Student-t prior with 3 degrees of freedom (24), a small sample of distributions were selected for investigation by the GLM to model change in cognitive test performance with age, based on prior data distributions for these tasks in healthy populations (10, 25, 26). These included the following likelihood functions: hurdle negative binomial distribution, beta distributions and zero-one inflated beta distributions. The following seven age-trend models were generated: (1) all subjects, (2) all males, (3) all females, (4) high educated males, (5) low educated males, (6) high educated females, (7) low educated females.

The posterior predictive distribution from each GLM was graphically compared to the observed sample distribution to assess model adequacy (27). More fine-grained model evaluation and model comparison was examined with Leave One Out (LOO) cross-validation (28), and the best fitting model was defined through examination of expected log posterior density. When comparing between models, higher expected log posterior density values indicate better fit.

From the best fitting model, posterior samples were derived from Markov Chain Monte Carlo simulation using the brms No-U-Turn Sampler (NUTS) to provide performance estimates by age for each demographic group (24). Four chains were completed (each run independently on a different central processing unit), each with 5,000 warmup iterations to calibrate the sampler, and 5,000 sampling iterations, yielding a total of 20,000 post-warmup posterior samples (29). Posterior samples were smoothed to avoid local minima in performance estimates with age. Recursive substitution was applied; in that if any estimated value for a given age is lower or higher than the value for the previous age (depending on the trend for the outcome measure), the estimated performance was substituted for the previous observation. Performance percentiles in 1% intervals were derived straight from the 20,000 posterior samples for each of the seven age-trend models.

Normative data are provided in the form of performance percentiles, with 50% reflecting average performance, above 50% reflecting above average performance, and lower percentiles reflecting poorer performance. For the purpose of providing normative comparison, percentiles for tied scores were calculated as the middle of the percentile range for each performance level, as previously recommended (13), rounded up to the nearest whole number. For a single tied test score relating to performance, for example, in the 88–99th percentile, 94th percentile was selected.

### Comparison with traditional methods of deriving normative data

Normative results from the Bayesian methodology described above were compared to two other established methods for deriving normative data: (A) the stratified method, and (B) the linear regression method.

For the stratified method, test results were stratified by educational level (high and low), sex (male and female), and as a function of age (into six roughly evenly spaced age groups: 18–24, 25–34, 35–44, 45–54, 55–64, 65–75). Following previously described methods (30), normative statistics for each outcome measure (Mean, SD) were determined based on the observed data per relevant subgroup. All individual variables in the cognition data were transformed into a scale with a mean of 0 and an SD of 1. Performance for each individual on each outcome measure was therefore converted into a *z*-score using the following equation [*Z_i_* = (observed score - mean score)/SD].

For the linear regression method (B), models were applied to assess the mean effects of age, sex (0 = male, 1 = female), and years in education (0 = low and 1 = high) on test performance. All variables were entered simultaneously into the regression model. Influential observations were identified by visual inspection of Cook's distance plots and values that were unacceptably high were removed and model refitted. *Z*-scores were calculated following methods described by Van der Elst et al. (30), using regression coefficients for each outcome variable. Each participant's predicted score was calculated using regression betas (predicted score = Intercept (0) + (Age*ß_age_) + (Sex*ß_sex_) + (Education*ß_education_). Residuals of each score were calculated (*e_i_* = observed score-predicted score), and standardized [*Z_i_* = *e_i_*/SD (residual)].

For outcome measures where higher scores denote poorer performance, the sign of the z-score was reversed (*z*-score = -*Z_i_*). These *z*-scores were then converted into cumulative percentiles using *z*-score look-up tables, with the 50th centile denoting average performance, <50th centile denoting below average performance, and >50th centile denoting above average performance.

### Sensitivity analysis: cross-sectional

Cross-sectional analyses tested for distributional differences between methods. A random subsample of 200 participants was taken from the original dataset using the “sample” function from the R base package. Normative percentile conversions for different methods were plotted using scatterplots to examine differences in distributions. Test performance in this subsample was categorised according to the three groups for each method: low to impaired range (<25th centile) average range (25th−75th centile) and in the high range (>75th centile). Frequency of these categorisations within the randomly generated subsample was then compared across the different normative data approaches using Fisher exact tests to identify whether different methodologies produced significantly different sensitivities for high and low performance ranges.

## Results

### Participants

**Figure 2:**
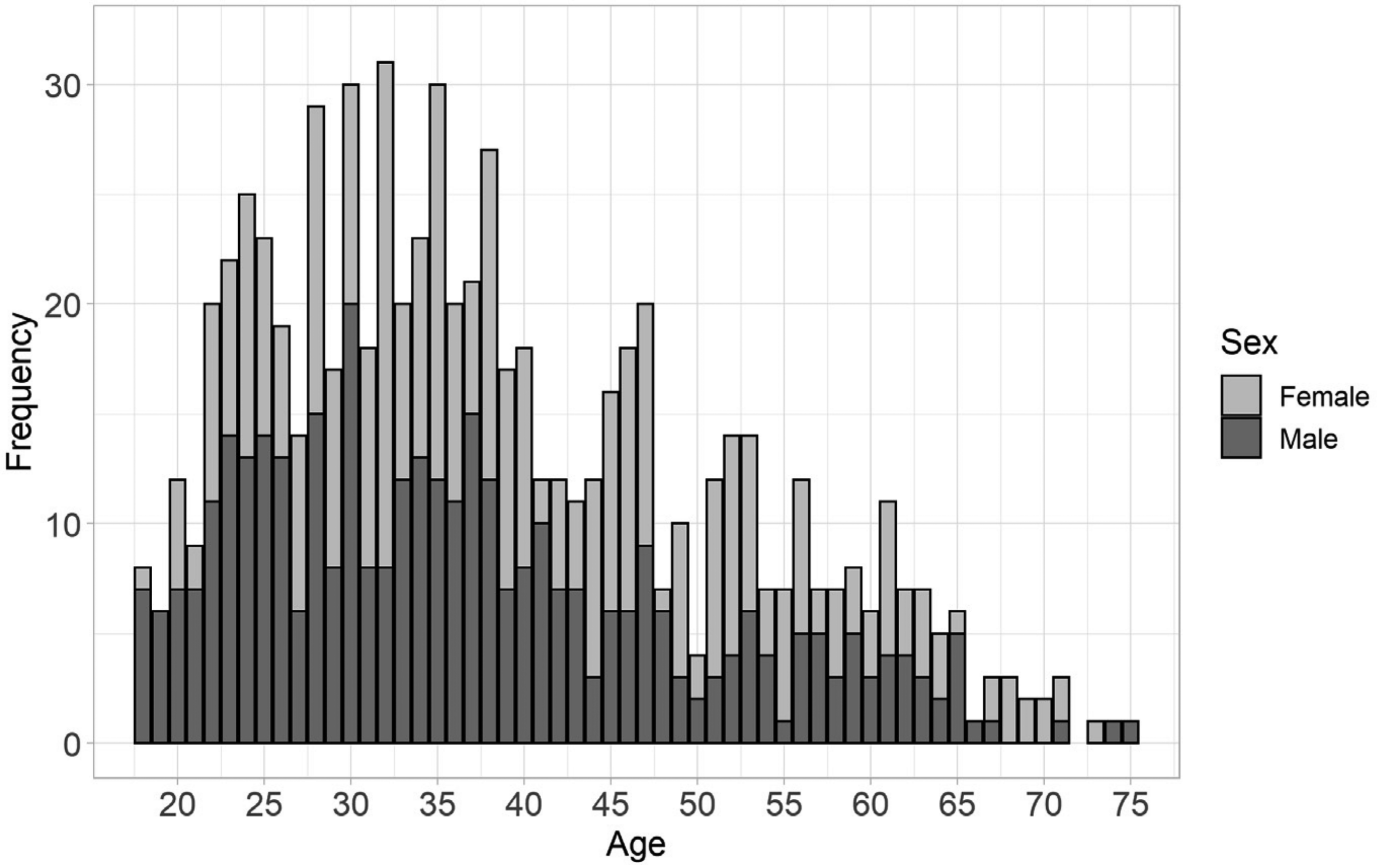
Stacked bar graph of distribution of ages represented in the participant sample by sex.

Data was obtained from 728 participants, primarily resident in the United Kingdom (*n* = 524), or the United States of America (USA, *n* = 110). The remainder resided in other countries in Europe (*n* = 60), Canada (*n* = 9), Australia (*n* = 5), Mexico (*n* = 2), Japan (*n* = 1) and Turkey (*n* = 1). All participants were fluent in English, with 628 reporting it to be their native language. The sample was sex-balanced with 368 male and 360 female participants. They were overall highly educated, with 65% (*n* = 470) having at minimum an undergraduate education, and only 15% (*n* = 102) completed their education at or before age 16. Mean age was 38.38 (median 36.0), ranging from 18 to 75 years. There was a high proportion of participants aged 40 and under (*n* = 459, 63% of the sample) with a smaller representation for those aged over 40 (Figure 2). Most participants completed assessments in full-screen mode (83%, *n* = 606).

### Bayesian analysis results

The best fitting likelihood distributions for all cognitive outcome measures in the Bayesian GLM were non-normal. Results from Bayesian models examining age coefficients in the different groups are presented in Table 1, alongside the likelihood distributions providing best fit for each outcome measure. These show a decline in performance with age for PAL and SWM, reflected in odds ratios (ORs) and Incidence Risk Ratios (IRRs) for age coefficients greater than one for error measures (PALTEA, SMBE) and efficiency of search strategy (SWMS). Age coefficient ORs and IRRs of less than one are seen for correct responses on PAL (PALFAMS), similarly reflecting a decline in performance with age. No significant effect of age is seen for RVP outcome measures (RVPA’ and RVPPFA).

**Table 1:** Age coefficients for Bayesian models in different sample groups.

Outcome measure	Bayesian models parameters and age coefficients within each sample [95% confidence intervals]								
	Model likelihood distribution	Coefficient	All subjects	Modelled by sex		Modelled by sex and education level			
				Male	Female	Male education		Female education	
						High	Low	High	Low
PAL First attempt memory score (PALFAMS)	Zero one inflated beta	Odds ratio	0.989 [0.986–1.000]	0.993 [0.986–1.000]	**0.986** [0.979–0.992]	**0.989** [0.981–0.998]	1.004 [0.992–1.016]	**0.985** [0.976–0.994]	**0.988** [0.978–0.997]
PAL total errors adjusted (PALTEA)	Zero one inflated beta	Odds ratio	**1.015** [1.004–1.019]	**1.011** [1.004–1.019]	**1.019** [1.011–1.027]	**1.013** [1.004–1.023]	1.004 [0.989–1.020]	**1.023** [1.012–1.034]	**1.016** [1.004–1.028]
RVP probability of false alarm (RVPPFA)	Zero one inflated beta	Odds ratio	0.999 [0.994–1.005]	1.003 [0.994–1.011]	0.996 [0.987–1.004]	1.002 [0.992–1.012]	1.005 [0.988–1.020]	0.996 [0.984–1.007]	0.995 [0.983–1.007]
RVPA A prime	Beta	Odds Ratio	0.999 [0.995–1.003]	1.000 [0.993–1.006]	1.000 [0.994–1.005]	1.000 [0.992–1.007]	1.002 [0.991–1.013]	0.999 [0.991–1.007]	1.001 [0.994–1.008]
SWM strategy (SWMS)	Hurdle negative binomial	Incidence Risk Ratio	**1.005** [1.002–1.007]	**1.005** [1.002–1.009]	1.003 [1.000–1.007]	**1.007** [1.002–1.011]	1.002 [0.995–1.008]	1.002 [0.997–1.007]	1.005 [1.000–1.009]
SWM between errors (SWMBE)	Hurdle negative binomial	Incidence Risk Ratio	**1.011** [1.005–1.016]	**1.011** [1.003–1.020]	**1.010** [1.003–1.017]	1.010 [0.999–1.020]	1.015 [1.000–1.030]	**1.013** [1.002–1.024]	1.010 [1.000–1.019]

Odds ratios and incidence risk ratios presented alongside 95% confidence intervals. In bold are values where 95% confidence intervals do not straddle 1, indicating a significant age coefficient.

**Figure 3:**
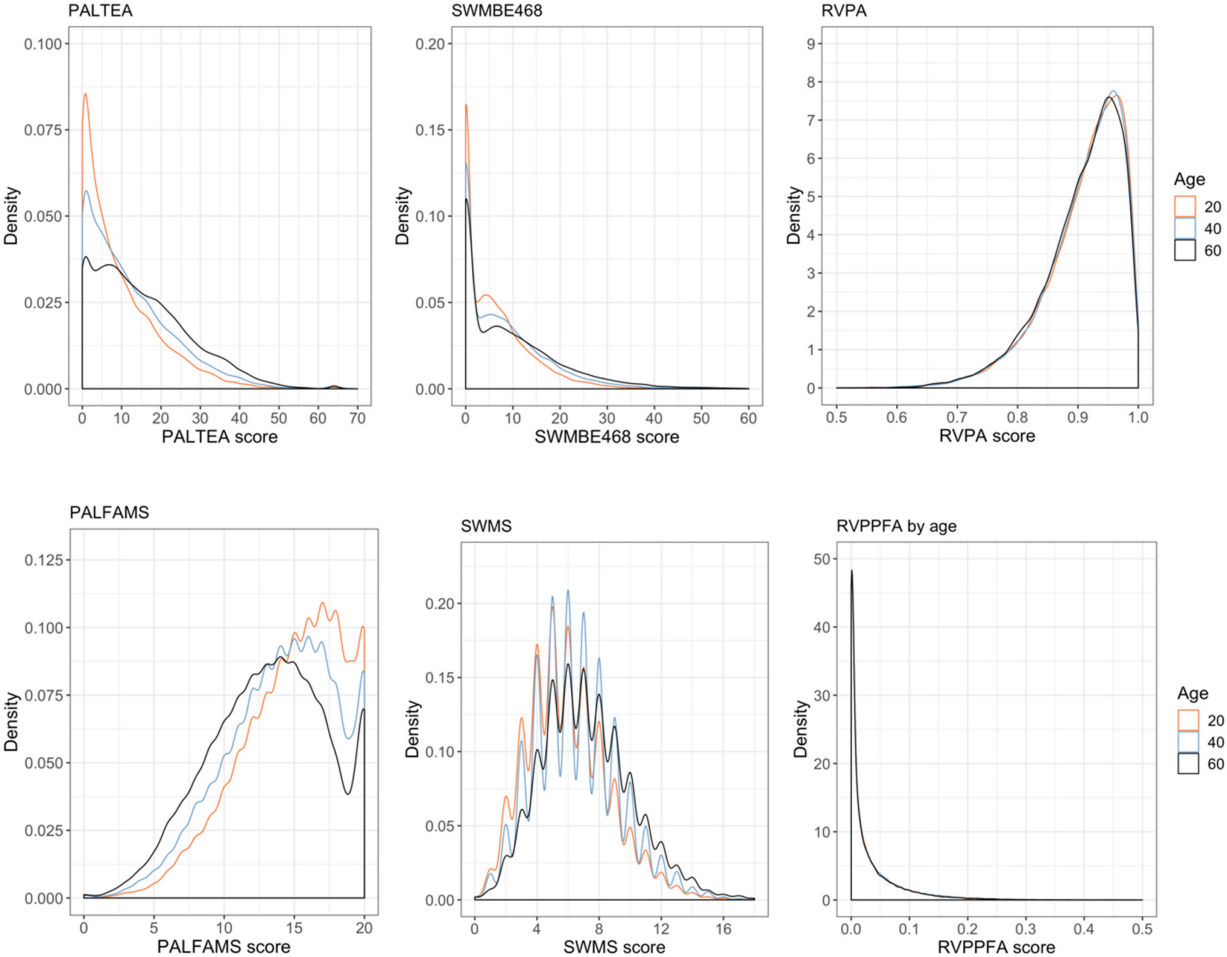
Posterior distributions for outcome measures by age, with raw scores on the *x*-axis and posterior distribution density on the *y*-axis, shown different colours for three age groups (orange = 20, blue = 40, and black = 60). Density scales are based on 20,000 predictions so sample size is fixed with no missing data. Range of density is determined by kernal (guassian) and bandwith used, and depends on the variance and scale of the outcome measure.

Posterior distributions generated by age are shown in Figure 3, showing age-related shifts in posterior distributions. For some measures these shifts are visually more apparent (e.g., PALTEA, PALFAMS and SWMBE), for other measures these are more subtle (SWMS) or not present (RVPA’, RVPPFA), in keeping with the strength of age coefficients shown in Table 1. The posterior distributions also highlight the non-normal distributions obtained for the cognitive test data.

**Figure 4:**
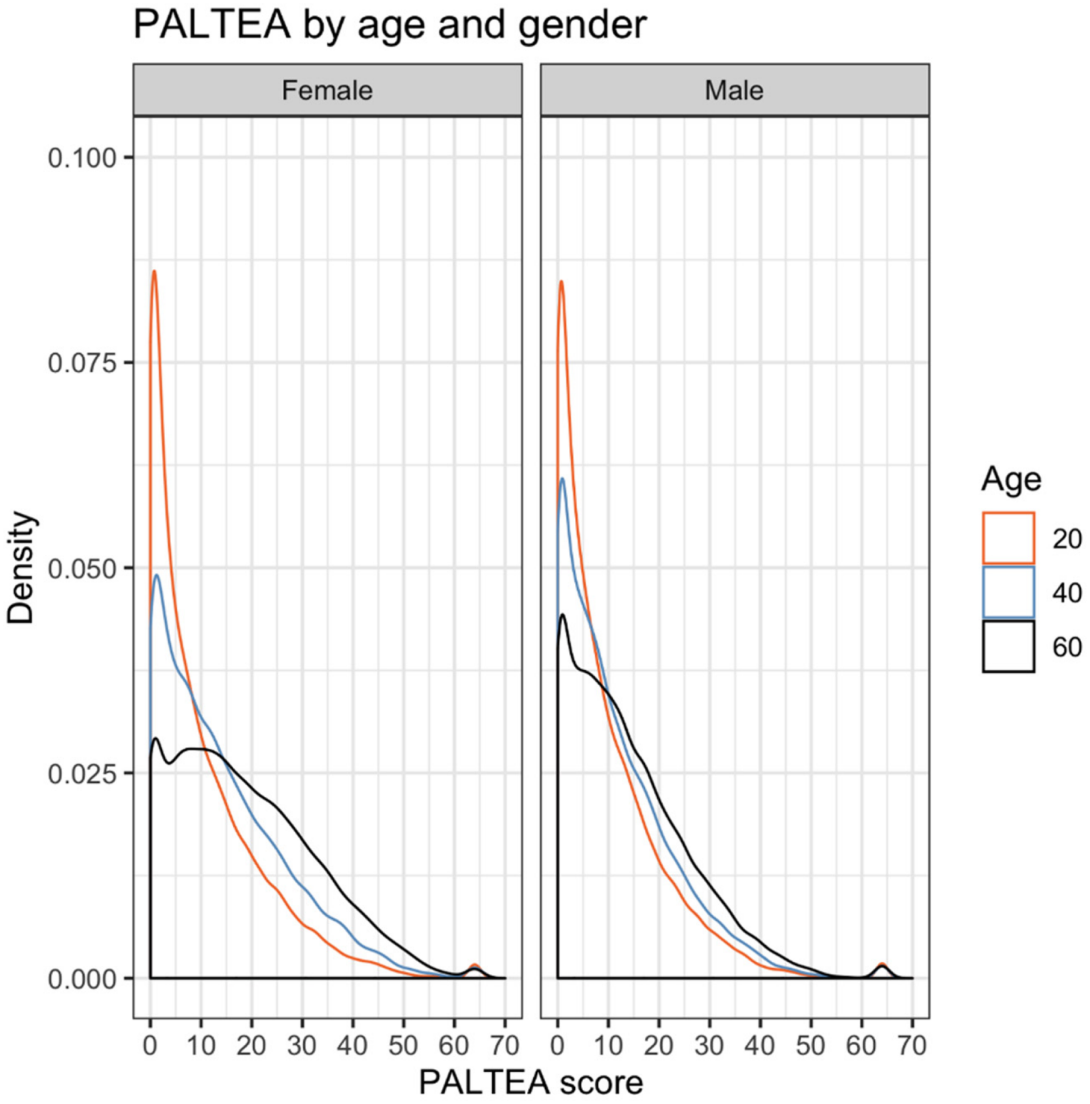
Posterior distributions for PALTEA by age and sex, with PALTEA raw scores on the *X* axis and posterior distribution density on the *Y* axis, shown different colours for three age groups (orange = 20, blue = 40, and black = 60).

**Figure 5:**
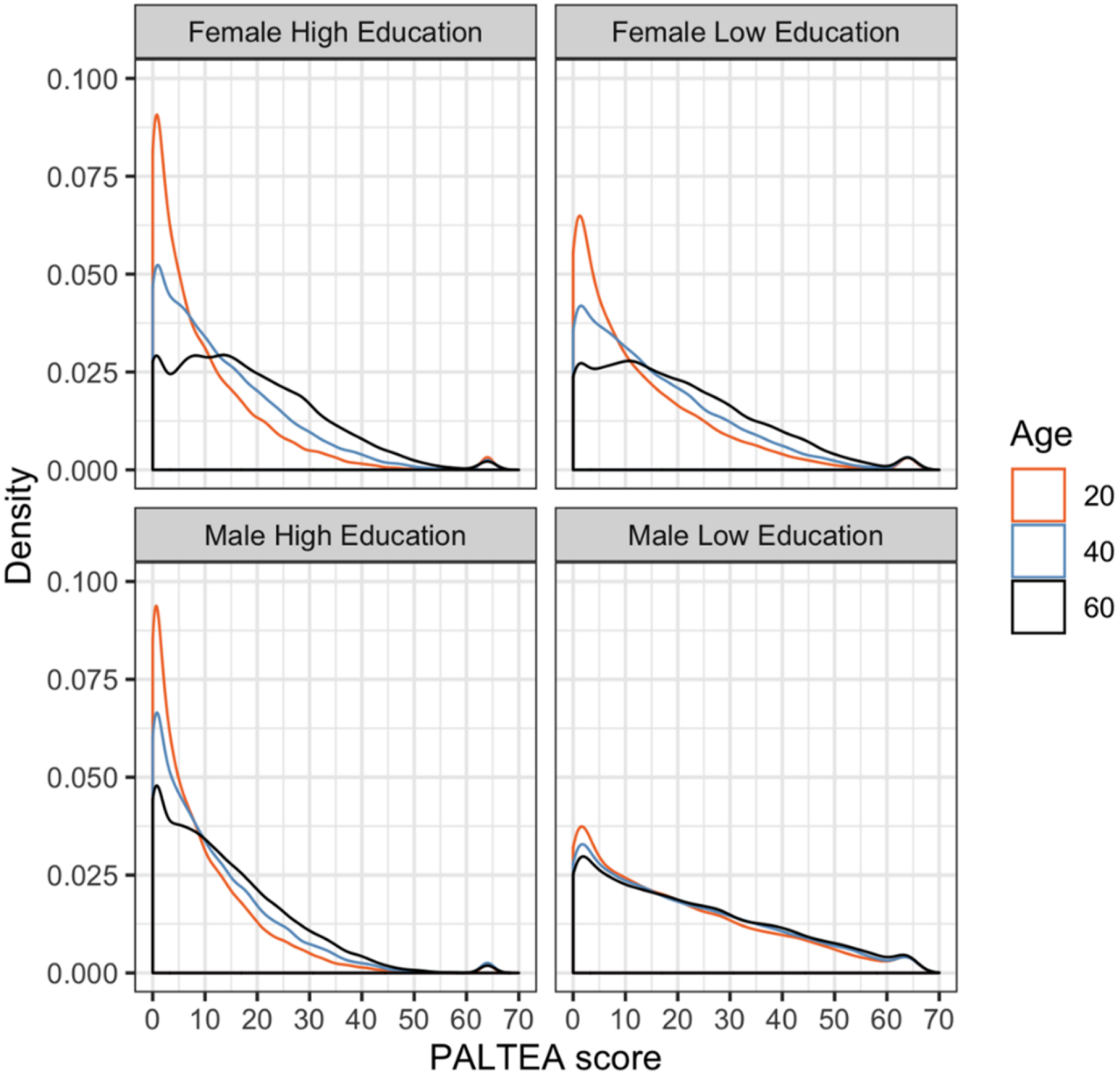
Posterior distributions for PALTEA by age, gender and education), with PALTEA raw scores on the *X* axis and posterior distribution frequency on the *Y* axis, shown different colours for three age groups (orange = 20, blue = 40, and black = 60).

Test performance differs by sex and education for several outcome measures. A visual overview of posterior distributions by sex and education categories is provided for PALTEA in Figures 4, 5, all others are provided in Supplementary Materials. Figure 4 shows a notable skew towards the zero errors for this task, particularly for younger participants, showing that many participants are performing at or around test ceiling. This skew towards zero errors remains present at older ages, albeit with a less extreme probability peak around zero, and a more breadth in response probability. Age effects are modestly more elevated for women on PALTEA, albeit not significantly so as shown by the overlapping confidence intervals for male and female groups (Table 1). Comparing PALTEA performance between women and men shows overall better performance of men than women across the lifespan, and with differences becoming more pronounced with older age, and with larger drops in performance with increasing age in women (Figure 3). Higher education is associated better PALTEA performance across all age ranges in both men and women (Figure 5), as indicated by modestly higher (albeit not statistically different) age coefficients within higher education groups. Reduced age differentiation is seen in men with a lower education, reflected in the non-significant coefficients for this model as seen in Table 1 (OR = 1.004, 95% confidence intervals: 0.989–1.020).

### Comparison with more traditional methods

Linear regression results are provided in Table 2. The direction of effect for linear models is complementary to those generated from the Bayesian approach. Linear regression identifies a significant effect of age for PAL and SWM outcome measures, but not for RVP. Measures show an increase in errors and a reduction in accurate responding with age. Educational level contributes to episodic memory test performance on PAL. No education effects are seen for the test of spatial working memory (SWM); however, this was the only test in which linear models identified sex differences, with strategy scores being significantly lower in males than females, showing better performance. Complementary results are generated in Bayesian analysis, where age coefficients are significantly elevated for men (and highly educated men in particular), but not for women. A discrepancy in results dependent on methodology is seen for results from the RVP. Using linear regression methods, educational level is associated with increased response sensitivity using the linear regression method. However, women and men with high education level are not found to perform better on the RVPA’ outcome measure using Bayesian analysis.

**Table 2:** Results from linear models of CANTAB outcome measures, intercept, regression coefficients and statistics for age, sex (male = 0, female = 1) and education level (low = 0, high = 1).

Outcome measure			Linear model predictors					
	Intercept		Age		Sex		Education level	
	Estimate (std error)	*t*-value	Estimate (std error)	*t*-value	Estimate (std error)	*t*-value	Estimate (std error)	*t*-value
PAL First attempt memory score (PALFAMS)	16.35 (0.54)	30.46***	−0.07 (0.01)	−5.82***	−0.03 (0.29)	−0.12	0.71 (0.31)	2.31*
PAL total errors adjusted (PALTEA)	5.039 (1.53)	3. 30**	0.20 (0.03)	6.16***	−0.10 (0.83)	−0.12	−2.31 (0.88)	−2.62**
RVP probability of false alarm (RVPPFA)	0.016 (0.004)	3.73***	0.0001 (0.0001)	−0.59	−0.0005 (0.002)	−0.21	−0.001 (0.003)	−0.51
RVPA A’	0.91 (0.009)	104.84***	−0.00003 (0.0002)	0.27	0.005 (0.005)	0.27	0.007 (0.005)	1.48*
SWM Strategy (SWMS)	5.18 (0.38)	13.73***	0.03 (0.008)	3.57***	0.57 (0.21)	2.74**	−0.01 (0.22)	0.96
SWM Between errors (SWMBE)	1.86 (1.15)	1.61	0.14 (0.02)	5.83***	0.82 (0.63)	1.30	−0.05 (0.67)	0.94

*** *p* < 0.001, **p < 0.01, *p < 0.05.

However, for all model residuals were non-normally distributed, showing either skewed distributions (floor: SWM Between errors, PAL total errors adjusted; ceiling: PAL first attempt memory score, RVP A’), or bimodal distributions (SWM strategy).

Stratified methods of deriving normative data provide similar overall results (see Supplementary Tables 1–6 for means, standard deviations and percentile ranges using age-, sex- and education-stratified groups). Results also indicate incremental deterioration in performance with increasing age. However, the results also show limits to the range of normative percentiles due to skew in the underlying datasets. The performance percentiles attainable by individuals with perfect scores vary significantly with age, with showing low percentile estimates (as low as <75th centile) for perfect test performance, particularly for younger participants.

### Sensitivity analysis: cross sectional comparison

Scatter plots comparing Bayesian vs. stratified and linear regression methods for deriving performance percentiles show positive relationship between methods. However, a curvilinear relationship between percentiles derived in these different ways is seen for certain outcome measures when comparing Bayesian methods to stratified and linear regression methods (Supplementary Figures 1–12). This was typically accentuated for younger participants with better task performance in tasks characterised by greater data skew. For these participants normative conversions were constricted more strongly within the top centiles using stratified and linear regression methods but allow a broader spread of percentile scores using Bayesian methods. This can be seen as an artifact of the high levels of ceiling performance in the younger groups, when normative data is generated through stratified and regression methodologies.

The number of participants classified to <25th, 25th–75th and >75th percentile groups was compared across each method (stratified, linear regression and Bayesian GLM) for each age category as defined using stratified normative data methods. The different methods used did not result in a statistically significant difference in number of participants allocated to each percentile group (<25th, 25th–75th and >75th) for most outcome measures (Supplementary Tables 7–9). In the 18–24 year age-group, different methods yielded a significant difference for SWM between errors (*p* = 0.02) within the subsample. More specifically, the Bayesian method typically classified participants more evenly across the percentile groups. This can be attributed, at least in part, to data skew, which limits the breadth of the distribution available for deriving normative ranges using stratified and linear regression methods. For example, for a male age 18–24 with a higher educational level the highest percentile score attainable on the SWM between errors is 72% using stratified methods and around 81% using linear regression methods. This limits allocation of participants to the higher performing group.

## Discussion

In the current study, a large but sparsely sampled health population was used to generate a large synthetic normative dataset capturing cognitive processes on the CANTAB. Bayesian methodology allowed modelling of non-normally distributed measures as a function of age, sex and education. Cross-validation of Bayesian and other well-established methodologies included age stratification where performance-based norms are derived from specific grouped age-ranges often spanning multiple years (31). In addition, the linear regression method which assumes equal rates of change in test performance across the lifespan (32). In a random subsample of 200 participants, it was established that the Bayesian approach showed a good degree of convergence with other established methods. However, it also allowed a broader spread of available normative data, by suppressing normative scores for participants performing at ceiling level to a lesser extent.

Whilst the Bayesian method developed in the current study performed comparably to other established methods, it also has clear benefits over the others. Normative data using stratification methods would require extremely large samples to incorporate sufficient variation at each stratum segregated by age, sex and educational level. As a result, normative data derived using these methods in the literature typically stratifies by broader age-groups, and infrequently by sex and educational level (31) since these tend to reduce restrict sample sizes which can introduce spurious variation. Gathering norms on a large-scale is crucial for understanding age-related changes in cognitive functioning. As the population changes, including an increasing aging population, the use of normative data will provide an accurate screening reference for clinical samples. The current findings on normative data are inconsistent with some indications that age, sex and education level influences performance whearas others have not found his association.

The current study indicates that sex and education may influence certain cognitive test profiles on CANTAB. These demographic measures may therefore be important to consider when considering the level of performance of a particular individual in relation to his or her peer group. Sex differences have been consistently documented in neuropsychological assessments and are in line with the findings from this study (33). Previous work with the CANTAB has shown better performance by men on measures of spatial working memory (26, 34) but less clear patterns for visuo-spatial episodic memory as measured by PAL (10, 35) and RVP (32, 34). Similarly, our findings of a decline in cognitive function increasing with age has been widely documented (36, 37). All methods for deriving normative data reflect a decline in cognitive function with age in tests of working and episodic memory (SWM and PAL, respectively), and no clear change with age for a measure of sustained attention (RVP). Similar age-related declines are reported in various countries using more typical laboratory-based assessments (32, 38, 39). Education does not appear to moderate the degree of age-related decline (40) with longer education consistently associated with improved cognitive functioning (41). Indeed, educational effects on the laboratory-based CANTAB assessments are well documented (32, 42, 43). Moreover, using stratified methods where performance measures are grouped for specific age-ranges (often spanning multiple years) (26), normative conversions may suffer from “age boundary” effects, due to more rapid cognitive change occurring during certain stages of life (i.e., in older age) (9). These “age boundary effects” flatten out normative estimates of cognitive functioning over the age-range during which performance has been aggregated and can result in in same raw scores artifactually producing very different normative scores when a participant crosses into another normative age group on later retesting. Normative data derived in this way may therefore be less suited to deliver the precision needed to differentiate between the normal aging process, clinically meaningful change and measurement artifact.

Generalisation of the study results is limited by the combined homogeneity and heterogeneity of its underlying population. The sample was homogeneous in its predominance of younger and more highly educated participants; however, similar recruitment biases have been identified in a number of web-based studies (44). Simultaneously, the sample is heterogeneous in its cultural and geographic breadth. As a study of web-based assessment collected using the assessment platform Prolific.co, it is likely to reflect the demographic of participants on this platform at the time where the study was carried out. Cross-regional differences in performance on CANTAB have been noted, where age and sex-stratified norms from developing countries tend to be lower than those from western populations and industrialised Asian countries (32). Due to limited samples from different demographic regions these aspects were not explored in the current study. It is also advisable to further examine socio-demographically diverse older adults which is a common limitation in remote studies that demonstrate high adherence but a homogenous sample of older adults (45). This is particularly important due to barriers such as social isolation which may hamper early detection of cognitive alterations. Care is likely required in the matching of the normative population to the population of interest, even when considering neuropsychological data acquired on web-based platforms. Another limitation may be that monitoring attention is not feasible with remote assessments. Nonetheless, reviews comparing remote to in-person studies have shown comparable results (46, 47) in clinical populations. It has also been recommended that deriving remotely collected norms will inform remote clinical assessments (48). Further studies comparing remote and in-person assessments, in particular regarding attention and particularly in diverse populations, are warranted.

## Data Availability

The original contributions presented in the study are included in the article/Supplementary Material, further inquiries can be directed to the corresponding author.
