# Supplementary material for: Generating normative data from web-based administration of the Cambridge Neuropsychological Test Automated Battery using a Bayesian framework
Source: Front Digit Health. 2024 Sep 20;6:1294222. doi: 10.3389/fdgth.2024.1294222 (PMC11451437; doi:10.3389/fdgth.2024.1294222)
Supplement: Supplementary file 1 [file Table1.docx]

**Section 1: Descriptives and Percentile ranges for outcome measures from stratified normative data approach**

|  | *female high education* | | | *female low education* | | | | *male high education* | | | *male low education* | | | |
| --- | --- | --- | --- | --- | --- | --- | --- | --- | --- | --- | --- | --- | --- | --- |
| Age  group | n | mean (sd) | %ile range | n | Mean (sd) | %ile range | n | | Mean (sd) | %ile range | n | Mean (sd) | %ile range |  |
| 18-24 | 15 | 7.33 (8.84) | 0-80 | 20 | 9.45 (7.86) | 0-89 | 49 | | 4.40 (7.27) | 0-73 | 16 | 6.38 (6.42) | 0-84 |  |
| 25-34 | 71 | 6.59 (7.81) | 0-80 | 36 | 6.22 (7.47) | 0-80 | 90 | | 6.56 (8.34) | 0-78 | 26 | 5.68 (6.22) | 0-82 |  |
| 35-44 | 61 | 8.62 (8.51) | 0-84 | 25 | 5.79 (6.96) | 0-80 | 71 | | 6.70 (9.07) | 0-77 | 21 | 11.10 (10.96) | 0-84 |  |
| 45-54 | 39 | 9.32 (10.80) | 0-81 | 32 | 9.09 (8.63) | 0-85 | 33 | | 8.82 (8.93) | 0-84 | 18 | 6.06 (7.36) | 0-80 |  |
| 55-64 | 18 | 8.33 (8.94) | 0-94 | 24 | 11.42 (8.12)) | 0-92 | 22 | | 10.27 (7.98) | 0-90 | 15 | 11.60 (10.20) | 0-87 |  |
| 65-75 | 7 | 18.29 (11.41) | 0-95 | 9 | 16.44 (6.89) | 0-99 | 7 | | 12.00 (6.76) | 0-96 | 3 | 6.67 (9.07) | 0-77 |  |

Supplementary table 1: SWMBE descriptives and percentile ranges from stratified normative data approach

|  | *female high education* | | | *female low education* | | | *male high education* | | | *male low education* | | |
| --- | --- | --- | --- | --- | --- | --- | --- | --- | --- | --- | --- | --- |
| Age  group | n | mean (sd) | %ile range | n | Mean (sd) | %ile range | n | Mean (sd) | %ile range | n | Mean (sd) | %ile range |
| 18-24 | 15 | 6.47 (2.29) | 0-97 | 20 | 7.15 (2.01) | 0-100 | 49 | 5.90 (2.50) | 0-94 | 16 | 5.75 (2.18) | 0-96 |
| 25-34 | 71 | 6.94 (2.48) | 0-98 | 36 | 6.67 (2.67) | 0-100 | 90 | 6.06 (2.98) | 0-91 | 26 | 5.92 (2.87) | 0-91 |
| 35-44 | 61 | 6.75 (3.00) | 1-94 | 25 | 6.00 (2.60) | 0-94 | 71 | 5.99 (2.95) | 0-91 | 21 | 7.00 (2.83) | 1-96 |
| 45-54 | 39 | 6.45 (3.18) | 1-92 | 32 | 7.28 (2.87) | 1-97 | 33 | 6.88 (2.77) | 0-96 | 18 | 5.56 (2.57) | 0-92 |
| 55-64 | 18 | 6.56 (2.53) | 0-96 | 24 | 7.54 (2.34) | 0-100 | 22 | 7.59 (2.72) | 1-98 | 15 | 6.67 (2.74) | 0-96 |
| 65-75 | 7 | 8.86 (2.80) | 3-99 | 9 | 8.78 (1.39) | 0-100 | 7 | 7.43 (2.15) | 0-99 | 3 | 4.33 (1.53) | 0-94 |

Supplementary table 2: SWMS descriptives and percentile ranges from stratified normative data approach

**Section 2: PAL descriptives and percentile ranges from stratified normative data approach**

|  | *female high education* | | | *female low education* | | | *male high education* | | | *male low education* | | |
| --- | --- | --- | --- | --- | --- | --- | --- | --- | --- | --- | --- | --- |
| Age  group | n | mean (sd) | %ile range | n | Mean (sd) | %ile range | n | Mean (sd) | %ile range | n | Mean (sd) | %ile range |
| 18-24 | 15 | 7.07 (6.34) | 0-87 | 20 | 10.25 (12.92) | 0-79 | 49 | 8.74 (11.98) | 0-77 | 16 | 15.19 (11.36) | 0-91 |
| 25-34 | 71 | 8.21 (8.52) | 0-83 | 36 | 12.08 (13.02) | 0-82 | 90 | 8.32 (11.97) | 0-76 | 26 | 11.73 (13.11) | 0-82 |
| 35-44 | 61 | 9.80 (11.38) | 0-81 | 25 | 14.24  (14.16) | 0-84 | 71 | 10.53 (9.87) | 0-86 | 21 | 11.19 (10.14) | 0-87 |
| 45-54 | 39 | 12.46 (11.56) | 0-86 | 32 | 11.78 (10.15) | 0-88 | 33 | 9.61 (9.40) | 0-85 | 18 | 11.61 (11.84) | 0-84 |
| 55-64 | 18 | 15.56 (11.62) | 0-91 | 24 | 16.36 (11.59) | 0-92 | 22 | 16.41 (12.96) | 0-90 | 15 | 18.87 (11.21) | 0-95 |
| 65-75 | 7 | 22.71 (20.41) | 1-87 | 9 | 20.56 (13.37) | 0-94 | 7 | 16.71 (13.38) | 0-89 | 3 | 6.67 (2.89) | 0-99 |

Supplementary table 3: PALTEA descriptives and percentile ranges from stratified normative data approach

|  | *female high education* | | | *female low education* | | | *male high education* | | | *male low education* | | |
| --- | --- | --- | --- | --- | --- | --- | --- | --- | --- | --- | --- | --- |
| Age  group | n | mean (sd) | %ile range | n | Mean (sd) | %ile range | n | Mean (sd) | %ile range | n | Mean (sd) | %ile range |
| 18-24 | 15 | 15.87 (2.88) | 0-93 | 20 | 14.50 (4.73) | 0-88 | 49 | 15.55 (3.90) | 0-87 | 16 | 12.31 (4.05) | 0-97 |
| 25-34 | 71 | 15.04 (3.88) | 0-90 | 36 | 14.08 (3.59) | 0-95 | 90 | 15.40 (4.11) | 0-87 | 26 | 14.08 (3.79) | 0-94 |
| 35-44 | 61 | 14.49 (3.74) | 0-93 | 25 | 13.48 (4.49) | 0-93 | 71 | 14.03 (3.95) | 0-94 | 21 | 14.29 (3.85) | 0-93 |
| 45-54 | 39 | 13.95 (3.64) | 0-95 | 32 | 13.84 (3.19) | 0-97 | 33 | 14.61 (4.10) | 0-91 | 18 | 14.17 (4.03) | 0-93 |
| 55-64 | 18 | 13.06 (3.70) | 0-97 | 24 | 12.33 (4.42) | 0-96 | 22 | 12.45 (4.47) | 0-95 | 15 | 12.13 (3.44) | 0-100 |
| 65-75 | 7 | 11.43 (4.86) | 1-96 | 9 | 11.11 (4.60) | 1-97 | 7 | 12.00 (3.79) | 0-98 | 3 | 16.67 (1.53) | 0-99 |

Supplementary table 4: PALFAMS descriptives and percentile ranges from stratified normative data approach

**Section 3: RVP descriptives and percentile ranges from stratified normative data approach**

|  | *female high education* | | | *female low education* | | | *male high education* | | | | *male low education* | | | |
| --- | --- | --- | --- | --- | --- | --- | --- | --- | --- | --- | --- | --- | --- | --- |
| Age  group | n | mean (sd) | %ile range | n | Mean (sd) | %ile range | n | Mean (sd) | %ile range | n | | Mean (sd) | %ile range |  |
| 18-24 | 15 | 0.88 (0.12) | 0-85 | 20 | 0.88 (0.09) | 0-92 | 49 | 0.93 (0.04) | 0-95 | 16 | | 0.90 (0.06) | 0-97 |  |
| 25-34 | 71 | 0.92 (0.05) | 0-96 | 36 | 0.91 (0.05) | 0-96 | 90 | 0.92 (0.07) | 0-87 | 26 | | 0.91 (0.06) | 0-93 |  |
| 35-44 | 61 | 0.91 (0.06) | 0-93 | 25 | 0.91 (0.06) | 0-93 | 71 | 0.90 (0.10) | 0-85 | 21 | | 0.88 (0.10) | 0-89 |  |
| 45-54 | 39 | 0.90 (0.08) | 0-88 | 32 | 0.90 (0.05) | 0-98 | 33 | 0.93 (0.04) | 0-94 | 18 | | 0.90 (0.11) | 0-82 |  |
| 55-64 | 18 | 0.92 (0.04) | 0-98 | 24 | 0.90 (0.05) | 0-97 | 22 | 0.93 (0.04) | 0-94 | 15 | | 0.91 (0.05) | 0-96 |  |
| 65-75 | 7 | 0.89 (0.03) | 0-100 | 9 | 0.94 (0.02) | 0-100 | 7 | 0.91 (0.07) | 0-90 | 3 | | 0.90 (0.09) | 0-88 |  |

Supplementary table 5: RVP A’ descriptives and percentile ranges from stratified normative data approach

|  | *female high education* | | | *female low education* | | | *male high education* | | | *male low education* | | |
| --- | --- | --- | --- | --- | --- | --- | --- | --- | --- | --- | --- | --- |
| Age  group | n | mean (sd) | %ile range | n | Mean (sd) | %ile range | n | Mean (sd) | %ile range | n | Mean (sd) | %ile range |
| 18-24 | 15 | 0.02 (0.02) | 0-82 | 20 | 0.05 (0.17) | 0-62 | 49 | 0.01 (0.02) | 0-67 | 16 | 0.01 (0.01) | 0-90 |
| 25-34 | 71 | 0.02 (0.06) | 0-62 | 36 | 0.01 (0.02) | 0-74 | 90 | 0.02 (0.04) | 0-66 | 26 | 0.01 (0.02) | 0-69 |
| 35-44 | 61 | 0.04 (0.14) | 0-61 | 25 | 0.03 (0.07) | 0-66 | 71 | 0.05 (0.12) | 0-66 | 21 | 0.04 (0.09) | 0-69 |
| 45-54 | 39 | 0.02 (0.05) | 0-62 | 32 | 0.02 (0.05) | 0-67 | 33 | 0.02 (0.06) | 0-63 | 18 | 0.06 (0.19) | 0-62 |
| 55-64 | 18 | 0.01 (0.02) | 0-74 | 24 | 0.02 (0.05) | 0-67 | 22 | 0.01 (0.01) | 0-79 | 15 | 0.03 (0.11) | 0-62 |
| 65-75 | 7 | 0.004 (0.002) | 0-97 | 9 | 0.01 (0.01) | 0-80 | 7 | 0.02 (0.05) | 0-67 | 3 | 0.002 (0.003) | 0-76 |

Supplementary table 6: RVPPFA descriptives and percentile ranges from stratified normative data approach

**Section 2: Scatter plots of percentiles between Bayesian, linear regression and traditional stratified methods**


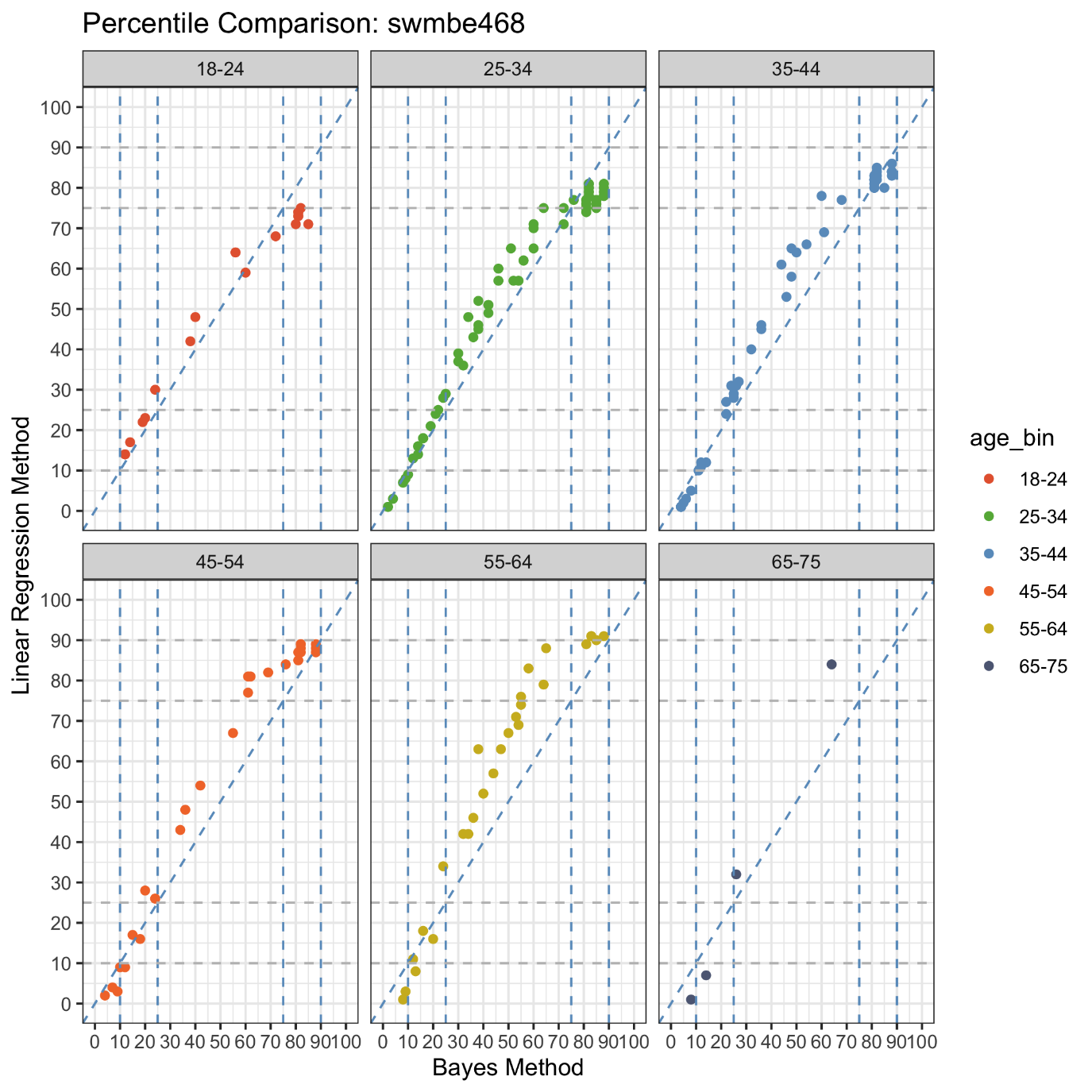


Supplementary figure 1: SWMBE scatterplot comparing percentile ranges from Bayesian and linear regression normative data approaches by age group


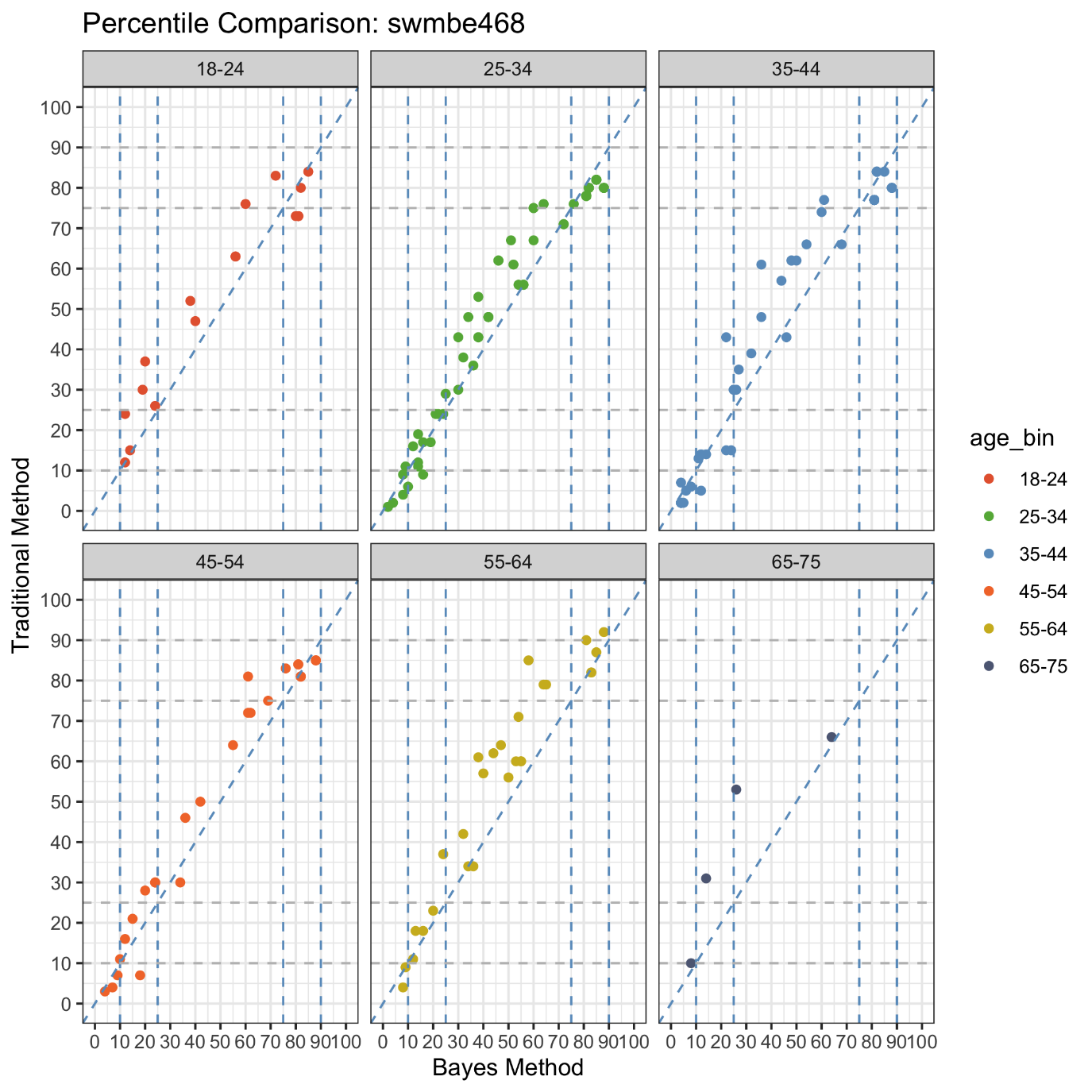


Supplementary figure 2: SWMBE scatterplot comparing percentile ranges from Bayesian and traditional stratified normative data approaches by age group


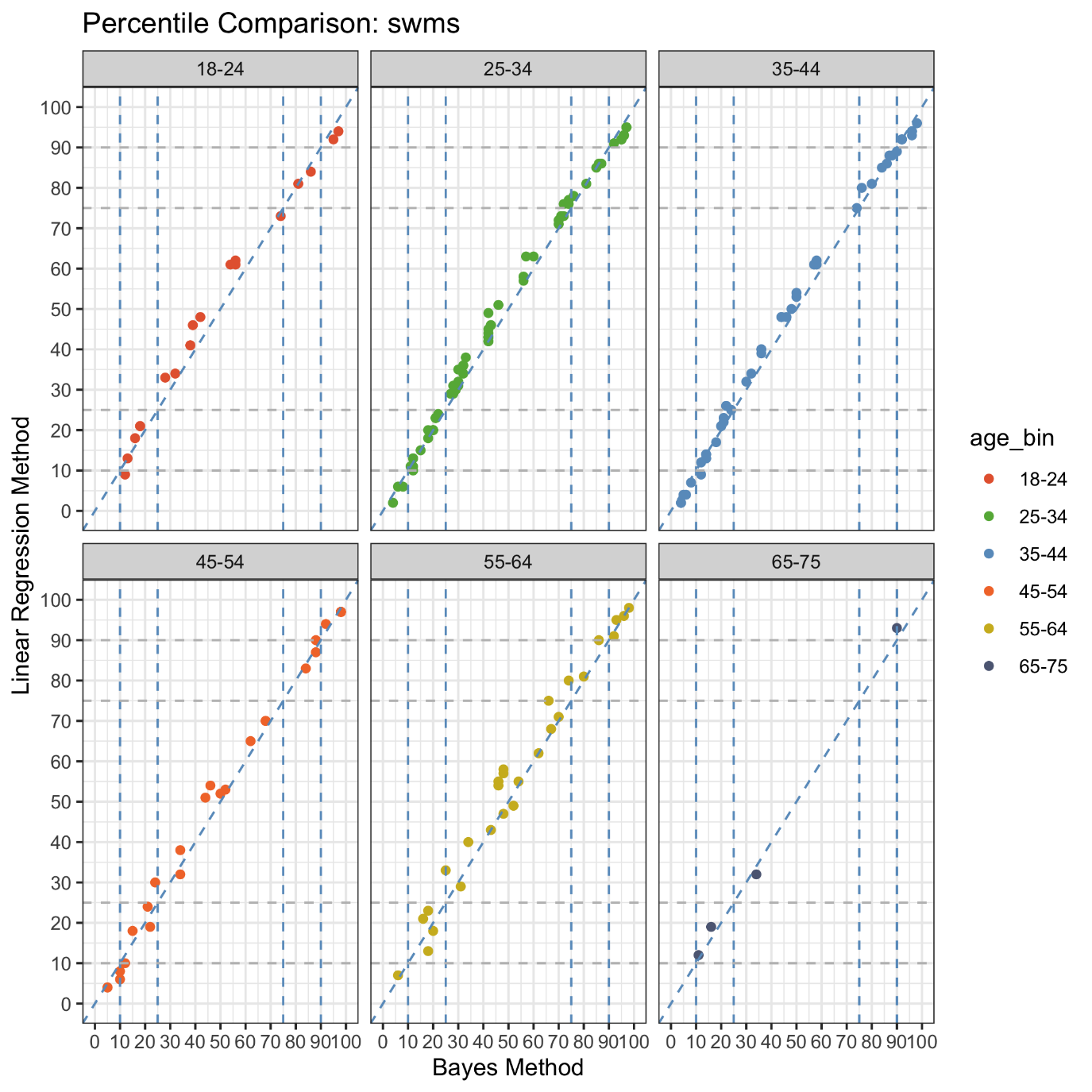


Supplementary figure 3: SWMS scatterplot comparing percentile ranges from Bayesian and linear regression normative data approaches by age group


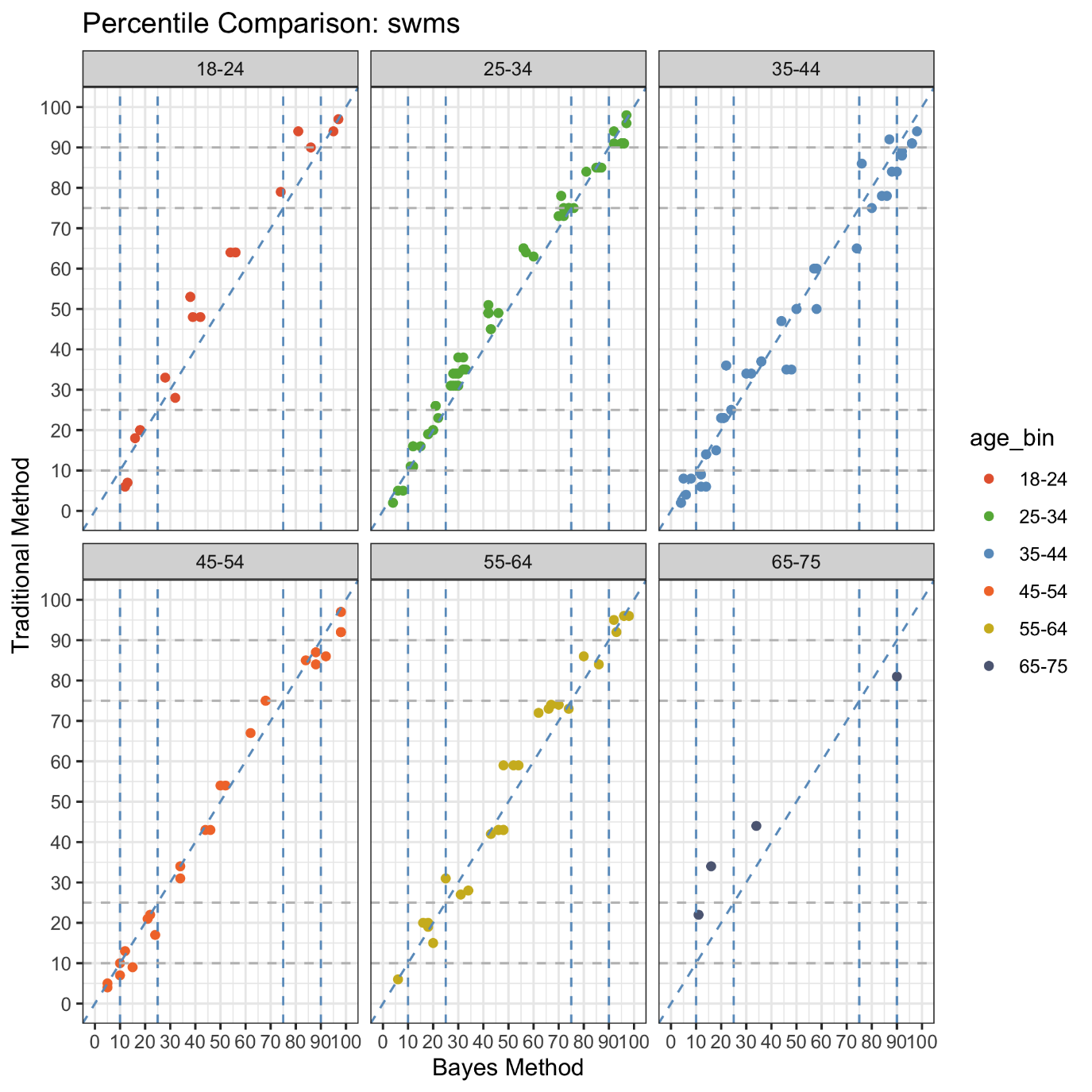


Supplementary figure 4: SWMS scatterplot comparing percentile ranges from Bayesian and traditional stratified normative data approaches by age group


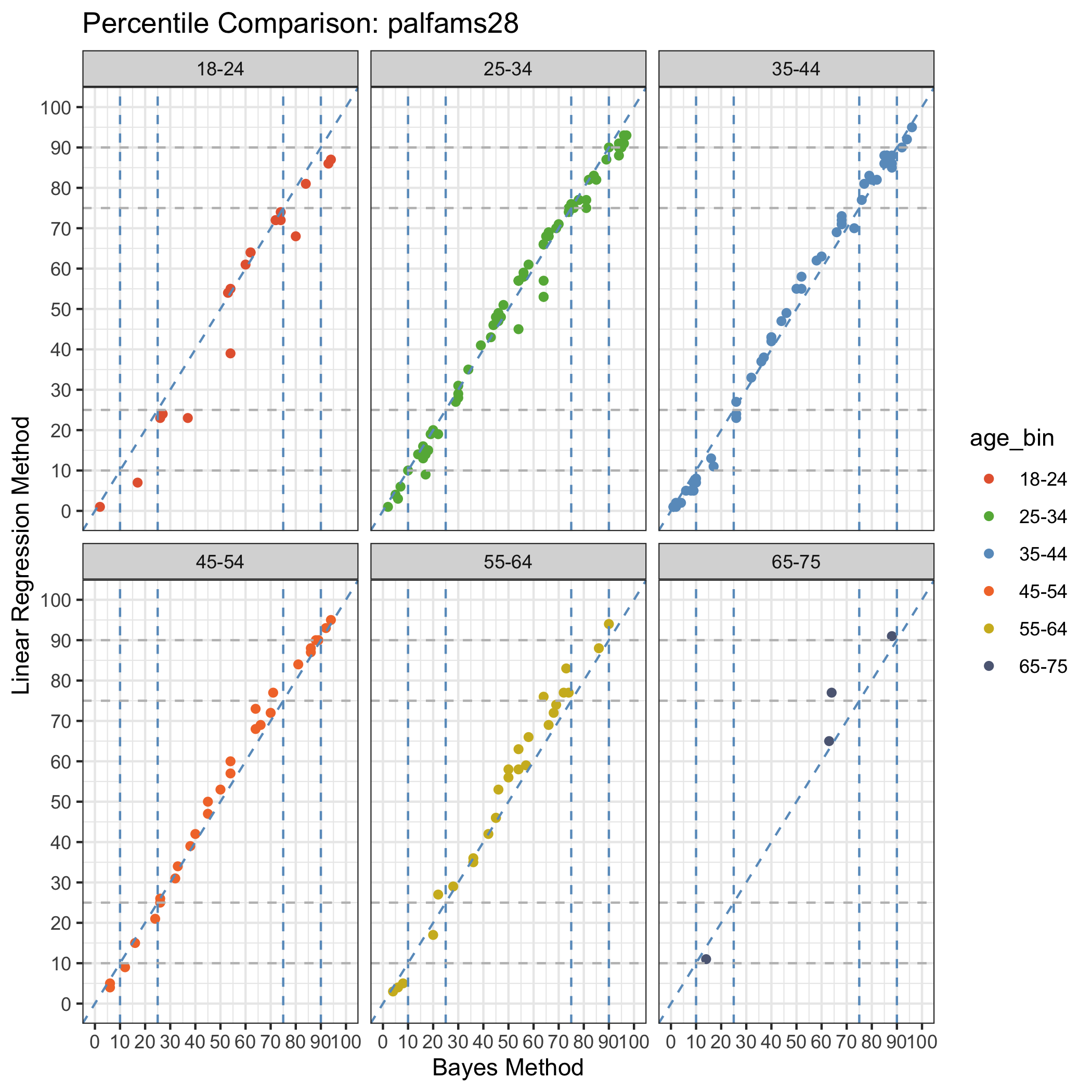


Supplementary figure 5: PALFAMs scatterplot comparing percentile ranges from Bayesian and linear regression normative data approaches by age group


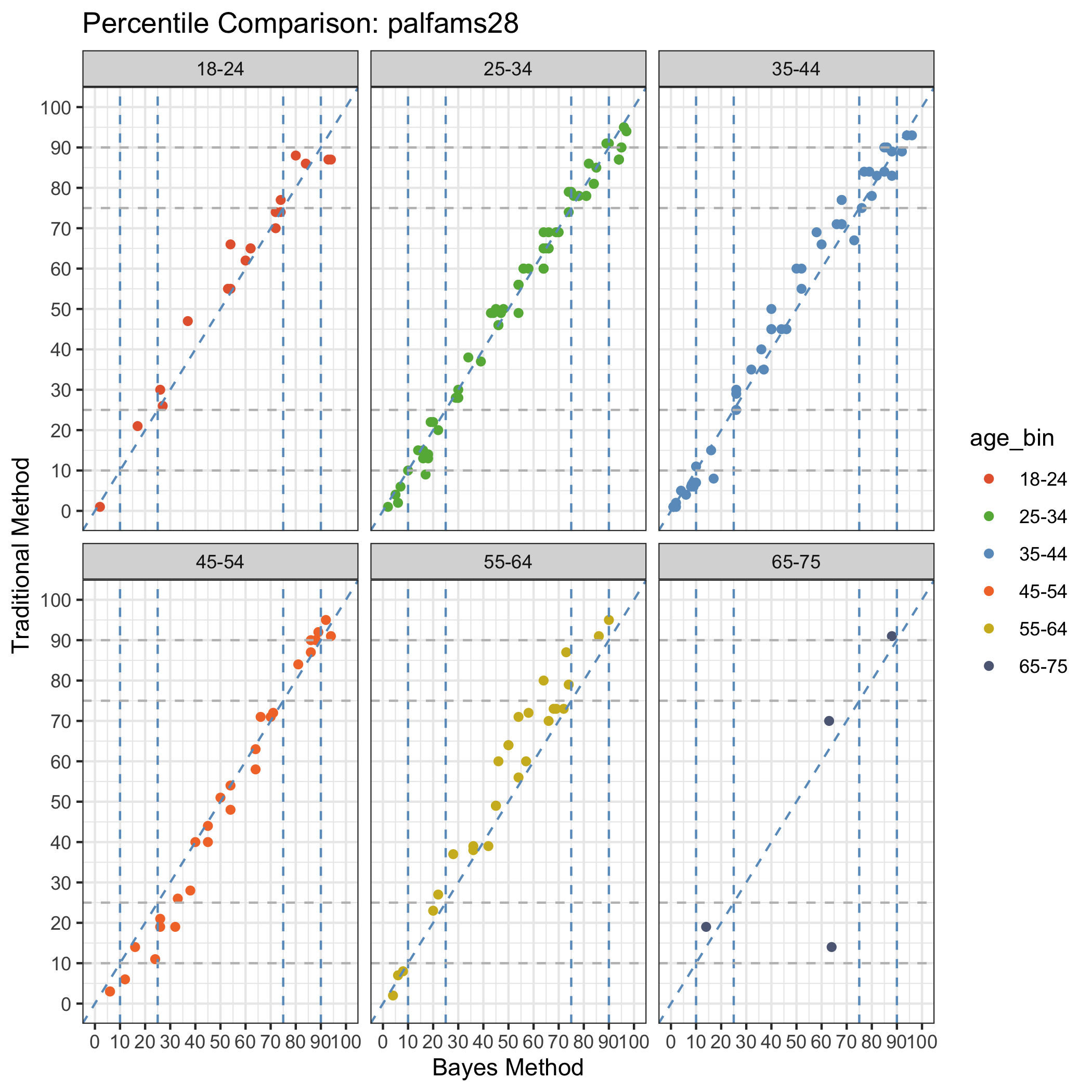


Supplementary figure 6: PALFAMs scatterplot comparing percentile ranges from Bayesian and traditional stratified normative data approaches by age group


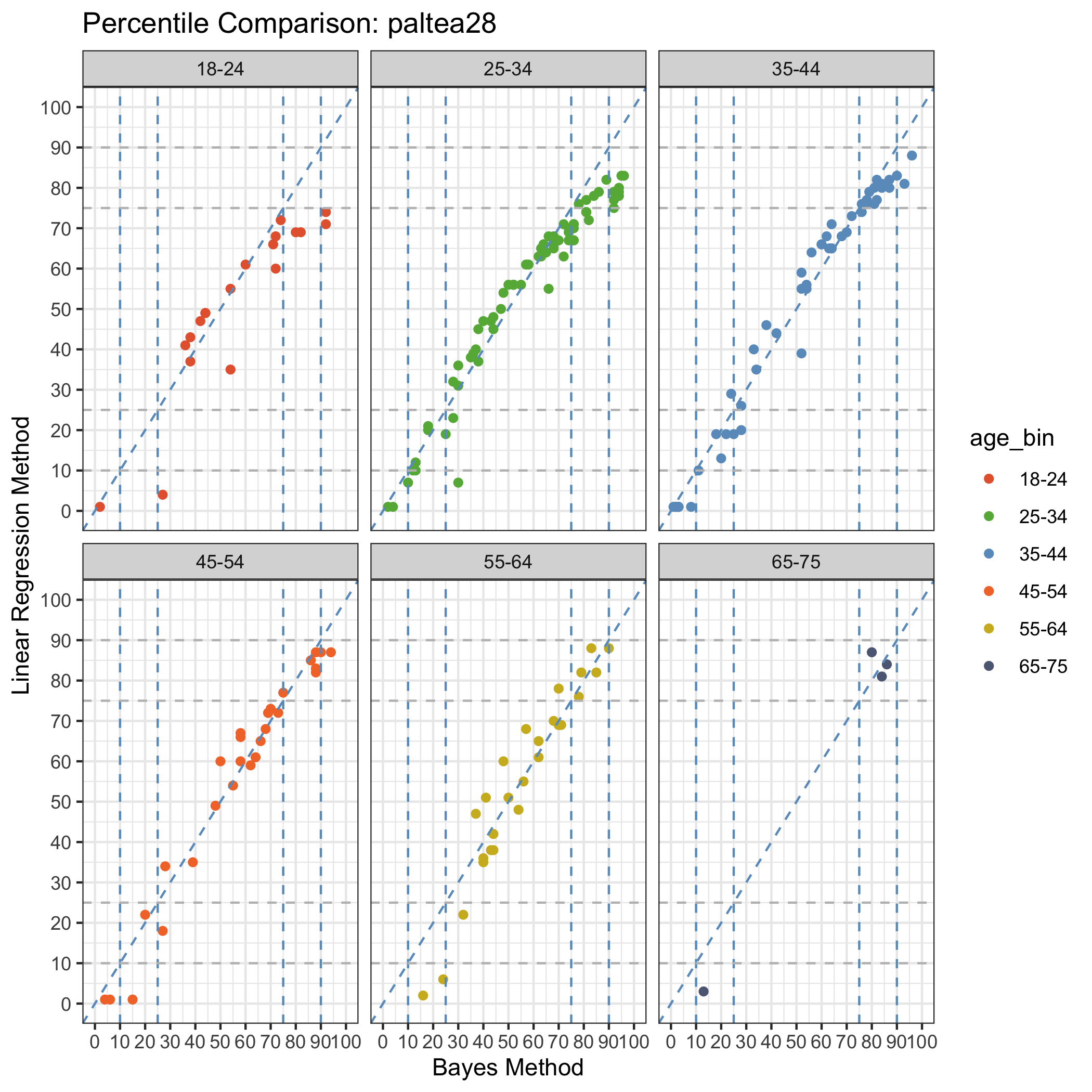


Supplementary figure 7: PALTEA scatterplot comparing percentile ranges from Bayesian and linear regression normative data approaches by age group


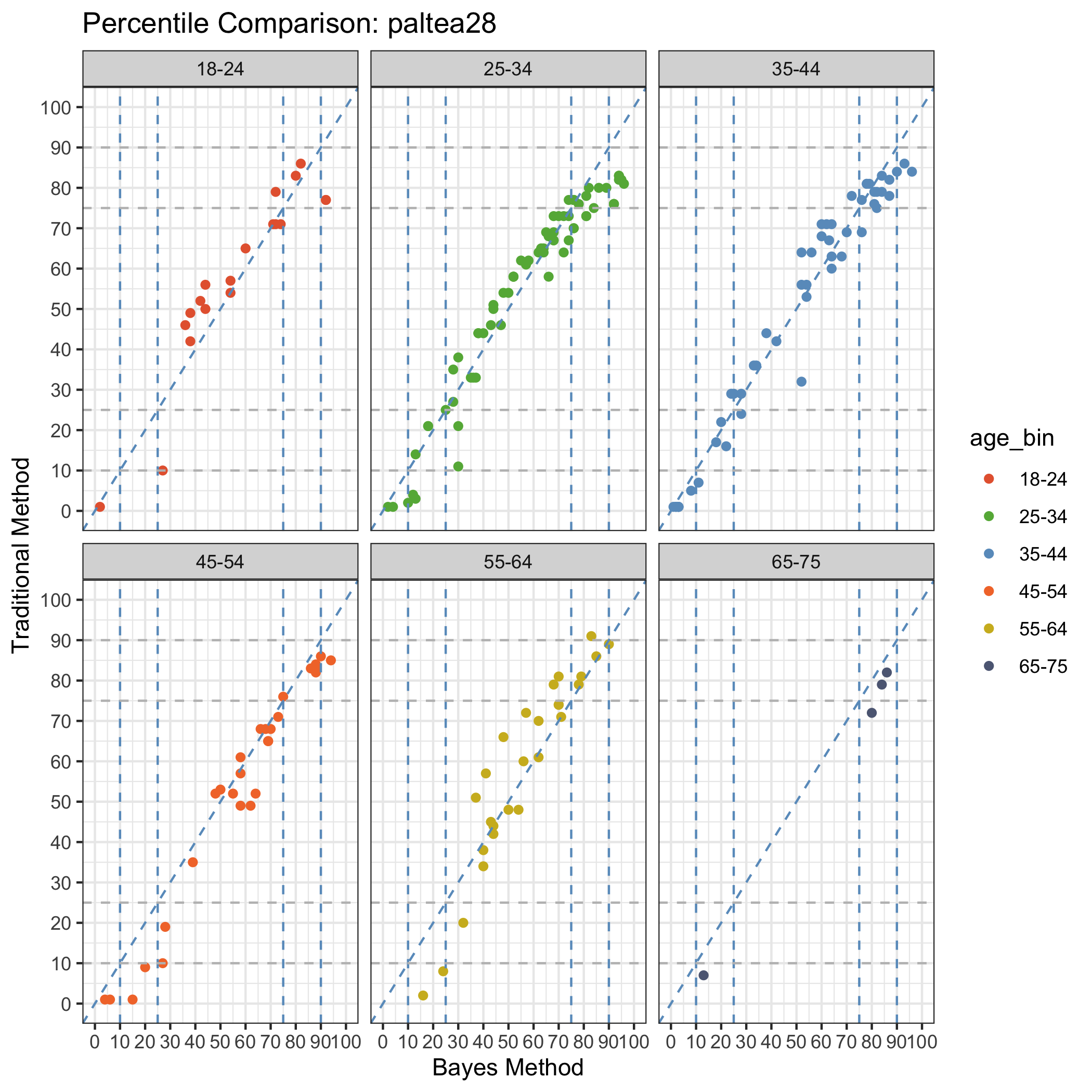


Supplementary figure 8: PALTEA scatterplot comparing percentile ranges from Bayesian and traditional stratified normative data approaches by age group


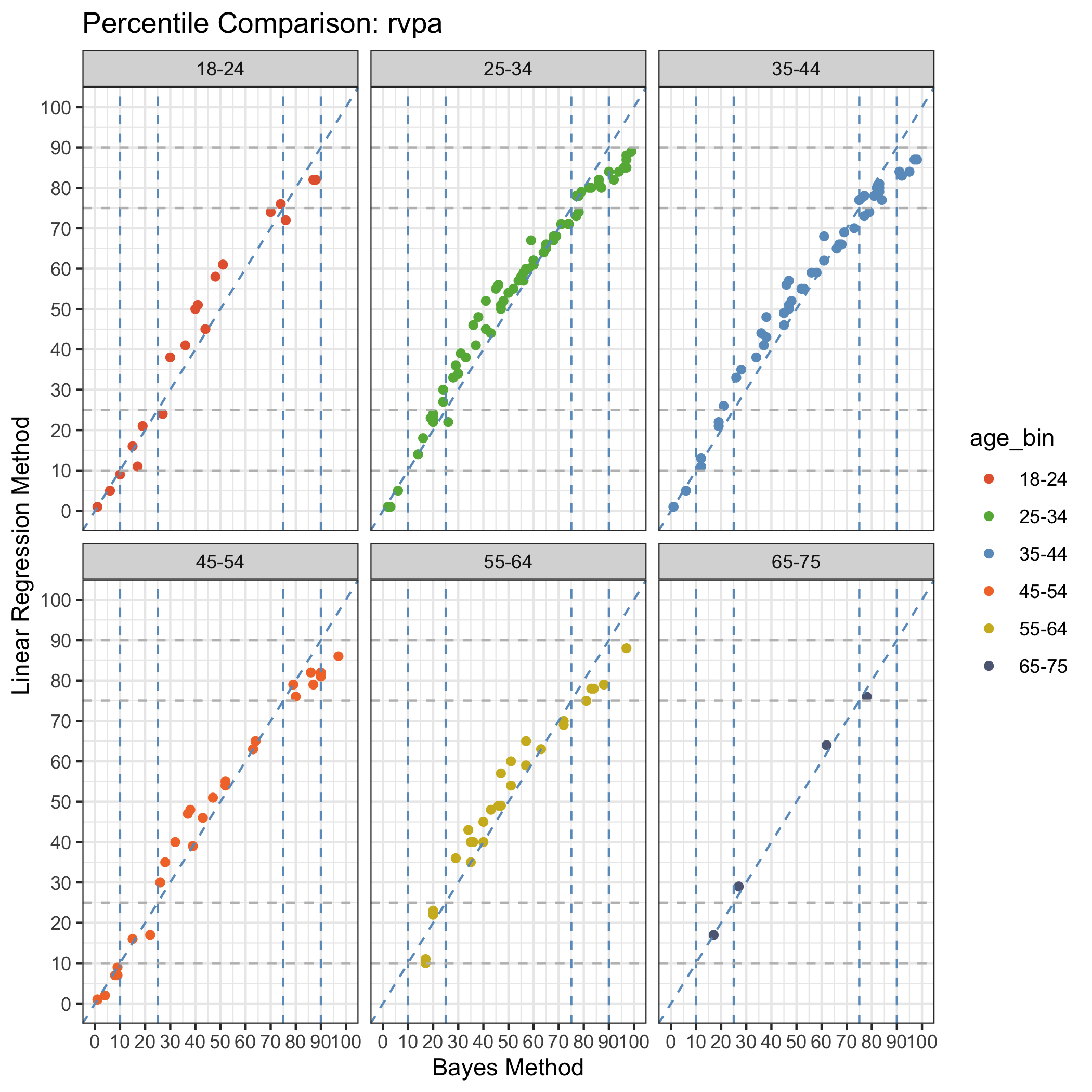


Supplementary figure 9: RVPA’ scatterplot comparing percentile ranges from Bayesian and linear regression normative data approaches by age group


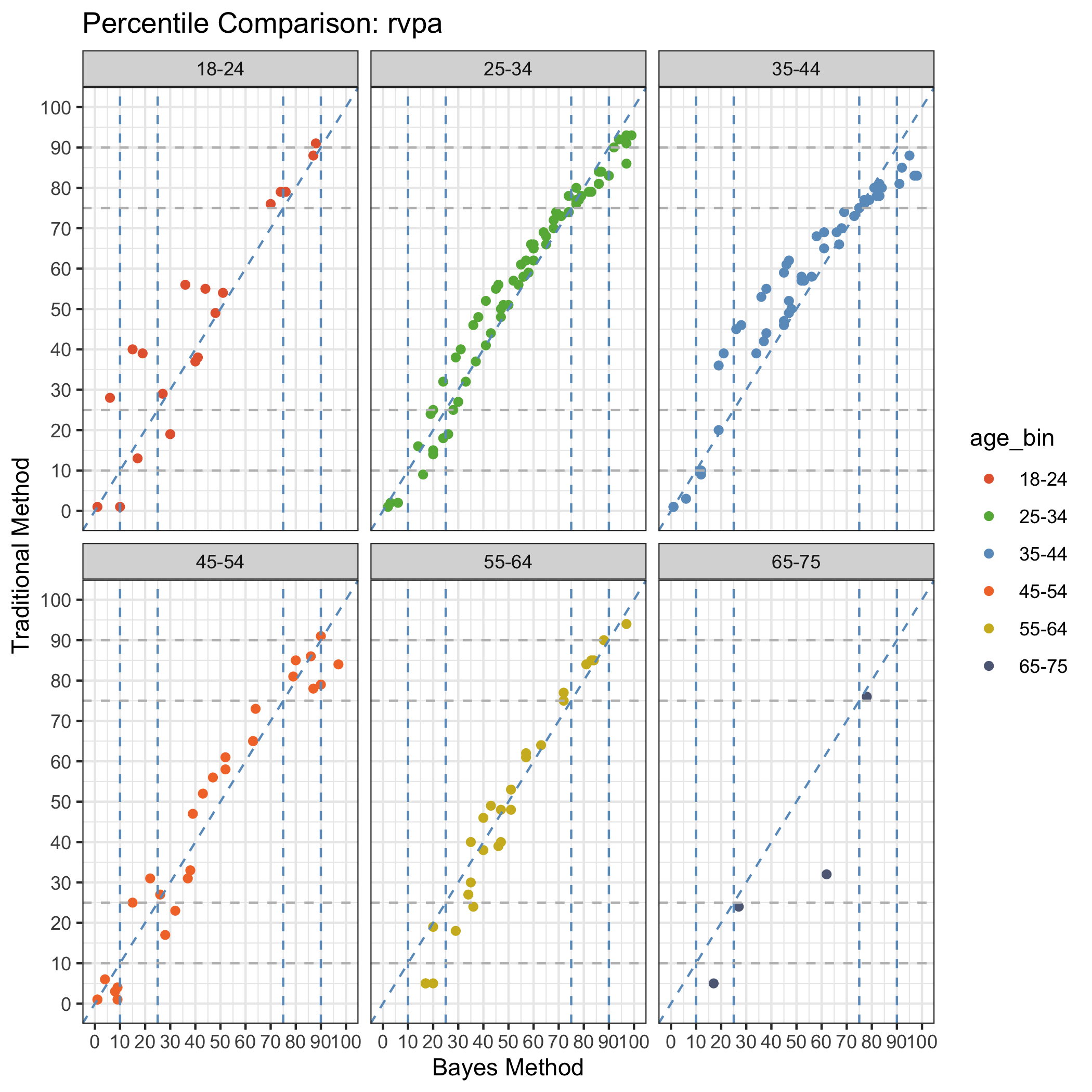


Supplementary figure 10: RVPA’ scatterplot comparing percentile ranges from Bayesian and traditional stratified normative data approaches by age group


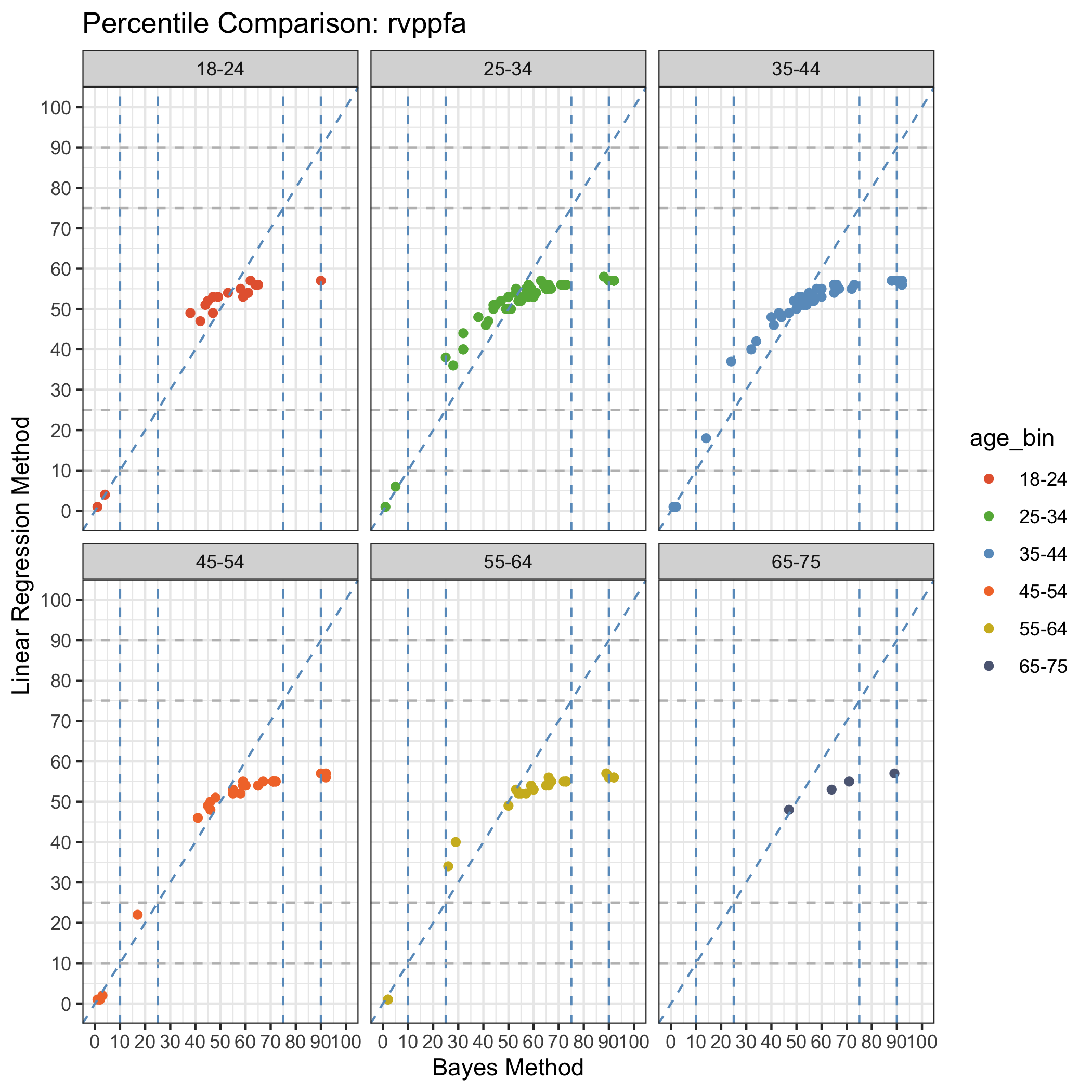


Supplementary figure 11: RVPPFA scatterplot comparing percentile ranges from Bayesian and linear regression normative data approaches by age group


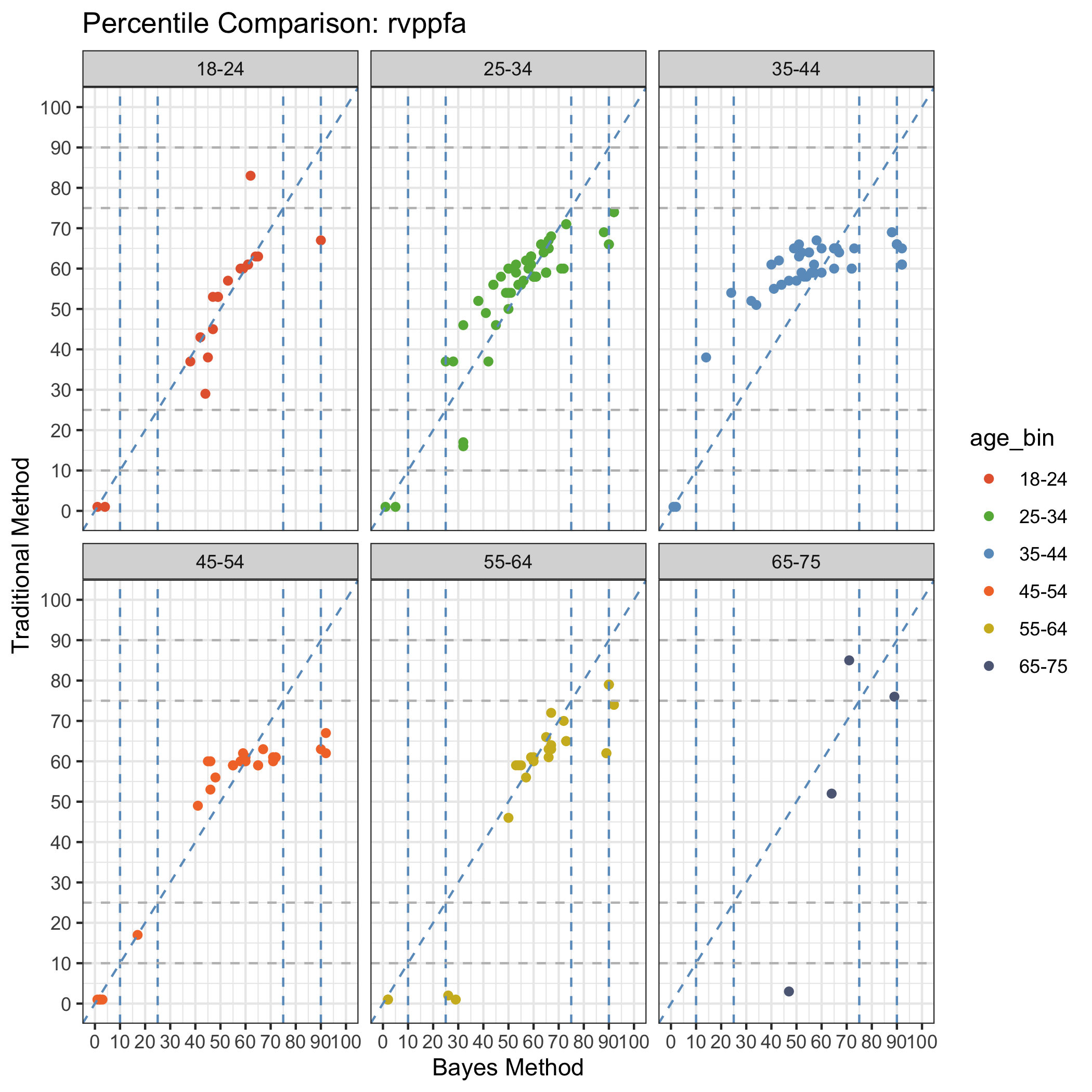


Supplementary figure 12: RVPPFA scatterplot comparing percentile ranges from Bayesian and traditional stratified normative data approaches by age group

**Section 3: Scatter plots of percentiles between Bayesian, linear regression and traditional stratified methods**

|  |  |  | PALTEA | | | | PALFAMS | | | |
| --- | --- | --- | --- | --- | --- | --- | --- | --- | --- | --- |
| Age | Number in subgroup | Percentile | Bayes method | Linear | Traditional | Fishers exact p-value | Bayes method | Linear | Traditional | Fishers exact p-value |
| 18-24 | 19 | 0-25 | 5.26 | 10.53 | 10.53 | 0.11 | 10.53 | 26.32 | 10.53 | 0.71 |
|  |  | 26-75 | 73.68 | 89.47 | 63.16 |  | 68.42 | 57.89 | 63.16 |  |
|  |  | 76-100 | 21.05 | 0 | 26.32 |  | 21.05 | 15.79 | 26.32 |  |
| 25-34 | 71 | 0-25 | 12.68 | 15.49 | 15.49 | 0.51 | 21.13 | 21.13 | 21.13 | 0.99 |
|  |  | 26-75 | 57.75 | 67.61 | 60.56 |  | 50.7 | 52.11 | 47.89 |  |
|  |  | 76-100 | 29.58 | 16.9 | 23.94 |  | 28.17 | 26.76 | 30.99 |  |
| 35-44 | 51 | 0-25 | 25.49 | 25.49 | 23.53 | 1 | 27.45 | 31.37 | 29.41 | 0.99 |
|  |  | 26-75 | 43.14 | 45.1 | 47.06 |  | 43.14 | 39.22 | 39.22 |  |
|  |  | 76-100 | 31.37 | 29.41 | 29.41 |  | 29.41 | 29.41 | 31.37 |  |
| 34-54 | 28 | 0-25 | 14.29 | 17.86 | 21.43 | 0.95 | 17.86 | 21.43 | 28.57 | 0.89 |
|  |  | 26-75 | 64.29 | 57.14 | 53.57 |  | 57.14 | 50 | 46.43 |  |
|  |  | 76-100 | 21.43 | 25 | 25 |  | 25 | 28.57 | 25 |  |
| 55-64 | 27 | 0-25 | 7.41 | 11.11 | 11.11 | 0.93 | 18.52 | 14.81 | 14.81 | 0.67 |
|  |  | 26-75 | 74.07 | 66.67 | 62.96 |  | 74.07 | 62.96 | 66.67 |  |
|  |  | 76-100 | 18.52 | 22.22 | 25.93 |  | 7.41 | 22.22 | 18.52 |  |
| 65-75 | 4 | 0-25 | 25 | 25 | 25 | 1 | 25 | 25 | 50 | 1 |
|  |  | 26-75 | 0 | 0 | 25 |  | 50 | 25 | 25 |  |
|  |  | 76-100 | 75 | 75 | 50 |  | 25 | 50 | 25 |  |

Supplementary table 7: PAL sensitivity analysis: frequency of allocation to percentile ranges (% of age group) according to different methods, alongside statistical comparison of allocation frequency

|  |  |  | RVPA’ | | | | RVPPFA | | | |
| --- | --- | --- | --- | --- | --- | --- | --- | --- | --- | --- |
| Age | Number in subgroup | Percentile | Bayes method | Linear | Traditional | Fischers exact p-value | Bayes method^a^ | Linear^b^ | Traditional^c^ | Fischers exact p-value |
| 18-24 | 19 | 0-25 | 31.58 | 36.84 | 21.05 | 0.82 | 10.53 | 10.53 | 10.53 | 1 |
|  |  | 26-75 | 52.63 | 47.37 | 52.63 |  | 84.21 | 89.47 | 84.21 |  |
|  |  | 76-100 | 15.79 | 15.79 | 26.32 |  | 5.26 | 0 | 5.26 |  |
| 25-34 | 71 | 0-25 | 15.49 | 14.08 | 16.9 | 0.9 | 4.23 | 2.82 | 5.63 | 0.001^a-b, a-c^ |
|  |  | 26-75 | 56.34 | 61.97 | 53.52 |  | 84.51 | 97.18 | 94.37 |  |
|  |  | 76-100 | 28.17 | 23.94 | 29.58 |  | 11.27 | 0 | 0 |  |
| 35-44 | 51 | 0-25 | 15.69 | 13.73 | 11.76 | 0.99 | 7.84 | 5.88 | 3.92 | <0.001 ^a-b, a-c^ |
|  |  | 26-75 | 56.86 | 58.82 | 60.78 |  | 68.63 | 94.12 | 96.08 |  |
|  |  | 76-100 | 27.45 | 27.45 | 27.45 |  | 23.53 | 0 | 0 |  |
| 34-54 | 28 | 0-25 | 28.57 | 28.57 | 32.14 | 1 | 17.86 | 17.86 | 17.86 | 0.32 |
|  |  | 26-75 | 46.43 | 46.43 | 42.86 |  | 71.43 | 82.14 | 82.14 |  |
|  |  | 76-100 | 25 | 25 | 25 |  | 10.71 | 0 | 0 |  |
| 55-64 | 27 | 0-25 | 14.81 | 14.81 | 22.22 | 0.85 | 7.41 | 7.41 | 14.81 | 0.07 ^a-b^ |
|  |  | 26-75 | 66.67 | 70.37 | 55.56 |  | 70.37 | 92.59 | 77.78 |  |
|  |  | 76-100 | 18.52 | 14.81 | 22.22 |  | 22.22 | 0 | 7.41 |  |
| 65-75 | 4 | 0-25 | 25 | 25 | 50 | 1 | 75 | 0 | 25 | 0.2 |
|  |  | 26-75 | 50 | 50 | 25 |  | 0 | 100 | 25 |  |
|  |  | 76-100 | 25 | 25 | 25 |  | 25 | 0 | 50 |  |

Supplementary table 8: RVP sensitivity analysis: frequency of allocation to percentile ranges (% of age group) according to different methods, alongside statistical comparison of allocation frequency.

^a-b^ significant post-hoc test between Bayesian and Linear methodologies

^a-c^ significant post-hoc test between Bayesian and Traditional methodologies

|  |  |  | SWMBE | | | | SWMS | | | |
| --- | --- | --- | --- | --- | --- | --- | --- | --- | --- | --- |
| Age | Number in subgroup | Percentile | Bayes method ^a^ | Linear^-b^ | Traditional | Fischers exact p-value | Bayes method^a^ | Linear^b^ | Traditional^c^ | Fischers exact p-value |
| 18-24 | 19 | 0-25 | 31.58 | 26.32 | 15.79 | 0.02^a-b^ | 10.53 | 10.53 | 10.53 | 1 |
|  |  | 26-75 | 31.58 | 73.68 | 63.16 |  | 84.21 | 89.47 | 84.21 |  |
|  |  | 76-100 | 36.84 | 0 | 21.05 |  | 5.26 | 0 | 5.26 |  |
| 25-34 | 71 | 0-25 | 25.35 | 22.54 | 23.94 | 0.61 | 4.23 | 2.82 | 5.63 | 0.92 |
|  |  | 26-75 | 33.8 | 45.07 | 33.8 |  | 84.51 | 97.18 | 94.37 |  |
|  |  | 76-100 | 40.85 | 32.39 | 42.25 |  | 11.27 | 0 | 0 |  |
| 35-44 | 51 | 0-25 | 33.33 | 25.49 | 27.45 | 0.93 | 7.84 | 5.88 | 3.92 | 0.98 |
|  |  | 26-75 | 27.45 | 31.37 | 31.37 |  | 68.63 | 94.12 | 96.08 |  |
|  |  | 76-100 | 39.22 | 43.14 | 41.18 |  | 23.53 | 0 | 0 |  |
| 34-54 | 28 | 0-25 | 35.71 | 25 | 25 | 0.74 | 17.86 | 17.86 | 17.86 | 1 |
|  |  | 26-75 | 28.57 | 25 | 35.71 |  | 71.43 | 82.14 | 82.14 |  |
|  |  | 76-100 | 35.71 | 50 | 39.29 |  | 10.71 | 0 | 0 |  |
| 55-64 | 27 | 0-25 | 25.93 | 22.22 | 22.22 | 0.83 | 7.41 | 7.41 | 14.81 | 1 |
|  |  | 26-75 | 55.56 | 44.44 | 48.15 |  | 70.37 | 92.59 | 77.78 |  |
|  |  | 76-100 | 18.52 | 33.33 | 29.63 |  | 22.22 | 0 | 7.41 |  |
| 65-75 | 4 | 0-25 | 50 | 50 | 25 | 0.77 | 75 | 0 | 25 | 1 |
|  |  | 26-75 | 50 | 25 | 75 |  | 0 | 100 | 25 |  |
|  |  | 76-100 | 0 | 25 | 0 |  | 25 | 0 | 50 |  |

Supplementary table 9: SWM sensitivity analysis: frequency of allocation to percentile ranges (% of age group) according to different methods, alongside statistical comparison of allocation frequency

^a-b^ significant post-hoc test between Bayesian and Linear methodologies
